# COP1 dynamics integrate conflicting seasonal light and thermal cues in the control of *Arabidopsis* elongation

**DOI:** 10.1126/sciadv.abp8412

**Published:** 2022-08-19

**Authors:** Cristina Nieto, Pablo Catalán, Luis Miguel Luengo, Martina Legris, Vadir López-Salmerón, Jean Michel Davière, Jorge J. Casal, Saúl Ares, Salomé Prat

**Affiliations:** ^1^ Centro Nacional de Biotecnologia (CNB), CSIC, Darwin 3, 28049 Madrid, Spain.; ^2^ Centro de Recursos Fitogeneticos y Agricultura Sostenible (CRF-INIA), CSIC, Autovia A2, km 32, 28805 Alcala de Henares, Madrid, Spain.; ^3^ Grupo Interdisciplinar de Sistemas Complejos (GISC), Madrid, Spain.; ^4^ Department of Mathematics, Universidad Carlos III de Madrid, Avenida de la Universidad 30, 28911 Leganes, Madrid, Spain.; ^5^ Centro de Investigación en Agrigenomica (CRAG), CSIC-IRTA-UAB-UB, 08193 Cerdanyola, Barcelona, Spain.; ^6^ Fundación Instituto Leloir, Instituto de Investigaciones Bioquímicas de Buenos Aires, Consejo Nacional de Investigaciones Científicas y Técnicas, 1405 Buenos Aires, Argentina.; ^7^ Instituto de Investigaciones Fisiológicas y Ecológicas Vinculadas a la Agricultura, Facultad de Agronomía, Universidad de Buenos Aires, Consejo Nacional de Investigaciones Científicas y Técnicas, 1417 Buenos Aires, Argentina.

## Abstract

As the summer approaches, plants experience enhanced light inputs and warm temperatures, two environmental cues with an opposite morphogenic impact. Key components of this response are PHYTOCHROME B (phyB), EARLY FLOWERING 3 (ELF3), and CONSTITUTIVE PHOTOMORPHOGENIC 1 (COP1). Here, we used single and double mutant/overexpression lines to fit a mathematical model incorporating known interactions of these regulators. The fitted model recapitulates thermal growth of all lines used and correctly predicts thermal behavior of others not used in the fit. While thermal COP1 function is accepted to be independent of diurnal timing, our model shows that it acts at temperature signaling only during daytime. Defective response of *cop1-4* mutants is epistatic to *phyB-9* and *elf3-8*, indicating that COP1 activity is essential to transduce phyB and ELF3 thermosensory function. Our thermal model provides a unique toolbox to identify best allelic combinations enhancing climate change resilience of crops adapted to different latitudes.

## Teaser

Using data for different light and temperature conditions, a model is proposed to optimize crops performance to climate change.

## INTRODUCTION

Light and temperature are key environmental factors that shape the pattern of plant growth according to prevailing environmental conditions. Growth of the hypocotyl, for instance, proceeds very rapidly in germinating *Arabidopsis* seedlings buried in soil to speed up exposure of photosynthetic organs to sunlight. Upon emergence, light severely reduces the hypocotyl growth rate, while growth is resumed on seedlings exposed to neighboring vegetation that threatens resource availability. Warm temperatures alike enhance hypocotyl growth to facilitate cooling of aerial tissues. The choice of sowing date is a critical decision in crop management to overlap the production phase with most favorable season. As sowing dates progress toward the warmer season, plants sense the increased light input of longer day lengths. However, how contrasting effects of light and temperature integrate to the control of thermomorphogenic development across seasons remains poorly understood.

The predominant photoreceptors inhibiting hypocotyl growth in *Arabidopsis* are phytochrome B (phyB), phyA, and cryptochromes. phyB acts as a main thermosensor, featuring a cross-talk interaction of light and temperature information already at the sensor level. phyB is synthesized in an inactive form (Pr) and photo-converted upon red light (R) perception into the active Pfr state that is translocated into the nucleus for light signal transduction (*1*). phyB senses changes in the R/FR ratio caused by neighboring plants due to far red light (FR) absorption by Pfr, leading to its conversion to Pr (*2*, *3*). Moreover, phyB Pfr slowly returns into the inactive Pr form, in a process termed thermal or dark reversion that is accelerated by warm temperatures (*4*, *5*). While phyA and cryptochromes have a main role in light-induced transcriptional reprogramming, so far there is no evidence for these photoreceptors acting as temperature sensors.

ELF3 confers the circadian clock’s “evening complex” (EC) thermal responsiveness via a prion-like thermosensory domain that reversibly directs protein phase transition (*6*). ELF3, ELF4, and LUX occupancy of their target loci is strongly reduced at warmer temperatures (*7*, *8*), with loss of *elf3*, *elf4*, or *lux* function leading to increased growth at 22°C, indicative of a constitutively activated thermal response in these mutants (*9*). Temperature-induced elongation is arbitrated by transcriptional activation of cell wall loosening and auxin biosynthetic/signaling genes by the PIF4 and PIF7 factors (*10*, *11*). Translation of PIF7 is increased at warm temperatures through structural changes of an RNA hairpin loop within the transcript 5′ untranslated region, with this control proposed to have a prevalent role on daytime thermomorphogenic pathway activation under longer day lengths (*11*).

Light-activated phyB suppresses PIF4 activity by signaling its phosphorylation and subsequent degradation by the 26*S* proteasome, in addition to prevent PIF4 binding to its target promoters (*12*, *13*). The circadian clock regulates rhythmic *PIF4* expression via transcriptional repression by the LUX, ELF4, and ELF3 proteins, comprising the core EC loop (*14*). The EC is recruited by the LUX factor to the *PIF4* promoter, suppressing *PIF4* expression during early night (*15*).

In addition, phyB Pfr inactivates the E3 ligase COP1, which acts as a photomorphogenesis suppressor in the dark. COP1 localizes into the nucleus in darkness, where it targets degradation of multiple photomorphogenesis-promoting factors, including LONG HYPOCOTYL IN FAR-RED 1 (HFR1) and ELONGATED HYPOCOTYL (HY5) (*16*–*18*). Red light–dependent phyB and SPA1 interaction dissociates the SPA1-COP1 complex and suppresses COP1 activity (*19*), besides triggering COP1 nuclear exclusion through a yet elusive mechanism (*20*). HFR1 and HY5 promote photomorphogenesis by antagonizing PIF4 through formation of inactive HFR1-PIF4 complexes and competitive interaction of HY5 with the PIF4 cognate elements (*21*–*24*). Elevated temperatures, on the other side, promote COP1 nuclear accumulation (*25*), alleviating HY5-suppressive effects. COP1 is also reported to trigger ELF3 destabilization during ELF3-dependent recruitment of GIGANTEA (GI) for COP1 degradation (*26*), although the relevance of this process in growth modulation remains unknown.

Together, these observations reveal that phyB, ELF3, PIF4, and COP1 form a thermal network where all these regulators physically and functionally interact with each other. Along with phyB suppression of PIFs and COP1 activity, COP1 is shown to facilitate phyB Pfr turnover under extended light (*27*). Furthermore, phyB favors ELF3 accumulation presumably by suppressing protein destabilization by COP1 (*28*), besides co-occupying at cooler temperatures many of the EC target loci (*7*). PIFs, in turn, promote COP1-mediated phyB degradation (*27*) and recruit phyB into the LIGHT_RESPONSE BTB1 and BTB2 (LRB1 and LRB2) complexes, implicated in phyB and PIF ubiquitination and mutual destruction (*29*, *30*). Thus, extensive connectivity of these regulators makes them prime candidates for modeling studies to dissect their exact roles in thermal responsiveness and assess whether their thermosensory function is dependent on light.

Here, we investigated the output of this network in young seedlings grown under different day lengths and 22°/28°C, as a mimic of seasonal information. We used the hypocotyl lengths of mutants and overexpression lines to fit a mathematical model based on the molecular interactions of these regulators. We validated the predicted molecular dynamics of these components and used the generated model to weigh their relative contribution to thermal elongation as influenced by day length.

## RESULTS

### Day length–dependent roles of phyB, ELF3, and COP1 in thermal elongation

**Fig. 1.**
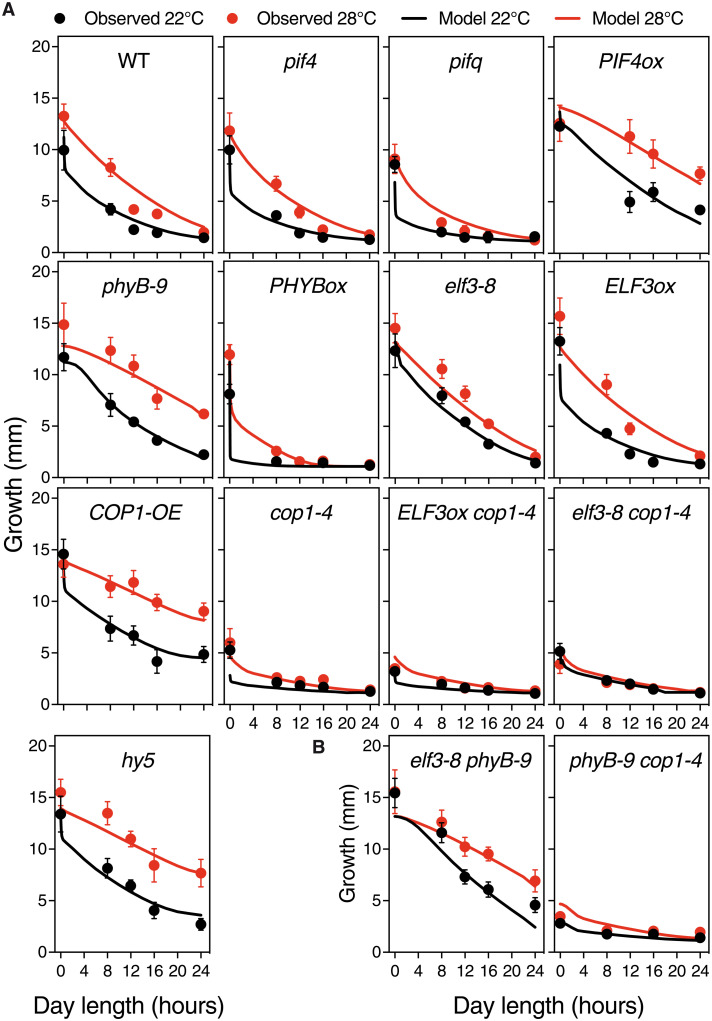
The model captures the growth data used for training and correctly predicts thermal growth of genotypes not used in the fitting process. (**A**) Hypocotyl lengths of the various *Arabidopsis* backgrounds grown at either 22° or 28°C and darkness, short days (8-hour light), 12-hour light, long days (16-hour light), and CWL. These data were used to train the mathematical model. Seeds were subjected to a 4-hour pulse of light to synchronize germination, and half of the plates were transferred to 28°C following seed germination. Seedlings were grown for 5 days, and plates were photographed for hypocotyl length measurement with ImageJ. Circles represent mean ± SD; the number of seedlings is indicated in table S2. Solid lines show the growth predicted by the trained model. The model accurately captures the main trends of data. WT, wild type. (**B**) Thermal growth behavior predicted for *elf3-8 phyB-9* and *phyB-9-cop1-4* mutants, not used to train the model. These two genotypes thus serve as a first validation step of the developed model. Symbols as in (A); the number of repetitions for each point is indicated in table S2. Shorter hypocotyls denoting later seed germination were not measured. In (A), values for *PHYB*ox 12-hour 22°C and *ELF3*ox 16-hour 28°C were skipped because of poor germination.

To gain a better understanding on the combined roles of phyB, ELF3, and COP1 in coordinating thermomorphogenesis, we examined temperature-induced growth of single and double mutants and overexpression lines in the Col-0 background (Fig. 1A). Light effects on thermal responsiveness were analyzed by measuring their hypocotyl lengths under continuous dark, continuous white light (CWL), and 8-, 12-, and 16-hour light cycles at a temperature of 22° or 28°C (Fig. 1 and Materials and Methods). In what follows, the terms “thermal responsiveness,” “thermal growth/elongation,” and “thermoelongation” are used indistinctly, always referring to the difference in hypocotyl length between 28° and 22°C.

Thermal response of wild-type plants was found to be inversely correlated with day length. In Col-0 seedlings, thermal elongation was greatest at 8-hour light and progressively reduced by 12- and 16-hour day lengths, while there was no thermal response in CWL (Fig. 1A). Thermal elongation was also substantially reduced in continuous darkness, suggesting that thermal response requires initial phyB Pfr photoconversion and photomorphogenic induction, in addition to alternating light/dark cycles. This is consistent with gating by the circadian clock to be pivotal to this response. Thermal growth was moreover suppressed in *pif4* and *pifq* mutants (Fig. 1A), while it was notably enhanced in *phyB-9* mutant and *PIF4*ox lines, in agreement with phyB acting at temperature perception (*4*) and a downstream role of PIF4 in promoting thermal elongation (*10*). However, as opposed to the inhibitory effects of increasing light hours in the wild-type and *pif* mutants, longer day lengths enhanced thermal responsiveness of the *phyB-9* and *PIF4*ox lines. These plants showed almost maximal thermoelongation in CWL, consistently with temperature inactivating phyB Pfr also during daytime (*5*).

Thermal response was impaired in all *cop1-4* genotypes, demonstrating that COP1 activity is essential to this response. These plants are much shorter in darkness than *pif4* and *pifq* mutants, in line with the strong constitutive photomorphogenic phenotype of this weak allele. Thermal sensitivity defects were also more severe than for *pif4* and *pifq* mutants, with *cop1-4* seedlings failing at thermal elongation, whereas *pif4* mutants are elongated in shorter day lengths. COP1 overexpression caused a similarly enhanced thermal response in CWL as seen in *phyB-9* mutants and *PIF4*ox lines, indicating that COP1 turnover of PIF-antagonizing factors like HY5 is pivotal to thermomorphogenic growth. A greater thermal response in CWL was also observed in the *hy5* mutant (Fig. 1A), consistent with targeted HY5 turnover being to a large extent responsible of this phenotype (*31*). *COP1*-*OE* lines were also significantly elongated in CWL, suggesting that COP1-dependent stabilization of PIF4 (*32*, *33*) is biologically more relevant in prolonged light.

Although PIF4 was reported to play a prominent role in driving thermomorphogenesis, *pif4* mutants showed a thermal response similar to the wild type in darkness or shorter day lengths (Tukey’s post hoc test; Fig. 1A), presumably due to PIF7 activity (*11*). Thermal sensitivity was, however, decreased in *pifq* mutants, indicating that PIF1, PIF3, and PIF5 contribute as well to thermal growth. Hypersensitive response to day length of *PHYB*ox lines is associated with suppressed thermomorphogenesis, indicating that thermal phyB Pfr inactivation leads to stabilization of all PIFs. Growth of *PHYBox* lines in darkness is almost identical to the wild type, consistent with all phyB being in the inactive Pr form and thus unresponsive to temperature.

As earlier reported, *elf3-8* mutants showed de-repressed thermal elongation at 22°C (*9*), although a residual response was still observed in all tested conditions (Tukey’s post hoc test; Fig. 1A). Unlike *PIF4*ox lines, growth of *elf3-8* mutants was inhibited in CWL, consistent with these plants showing a hypersensitive response to light, dependent on phyB turnover. ELF3 is a central component of the EC, which is shown to bind and suppress expression of many growth-regulating genes in addition to *PIF4* (*7*, *15*, *34*). In this regard, *ELF3*ox lines were impaired in thermal elongation in long days (16-hour light), although they behaved as the wild type in other diel conditions. These plants were as tall in darkness as *elf3-8* mutants, showing that interaction with phyB Pfr is key to ELF3 function (*35*).

**Fig. 2.**
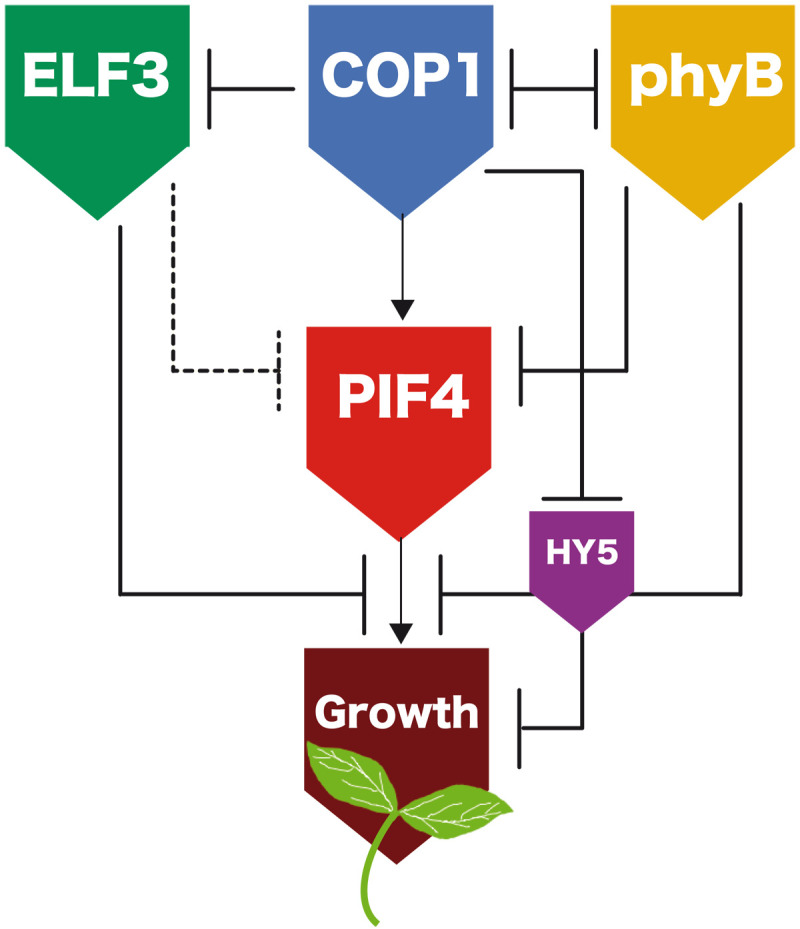
ELF3, COP1, phyB, PIF4, and HY5 form a highly connected network that controls hypocotyl growth. Schematic representation of the known interactions of these regulators and the model network. PIF4 activates the expression of cell wall loosening and auxin biosynthesis/responsive genes to initiate hypocotyl growth. ELF3, COP1, phyB, and HY5 regulate *PIF4* transcription, PIF4 protein stability, and binding of this factor to its cognate promoter elements. Dashed and solid lines respectively indicate transcriptional and posttranscriptional control as included in the model.

Together, these data reveal that phyB, COP1, and ELF3 govern thermal growth under diel conditions in a highly intertwined fashion (Fig. 2). Dynamic changes in phyB Pfr levels resulting from external light and temperature cues drive thermal elongation by suppressing PIF nuclear accumulation, along with COP1-mediated turnover of HY5 and presumably ELF3. On the other hand, thermal elongation of *phyB-9* and *PHYB*ox lines in the dark reveals that ELF3 and possibly COP1 have a role in modulating thermal responsiveness, independently of phyB Pfr.

### A mathematical model on activity of these regulators captures thermal growth of all genotypes

To assess biological significance of these events, measured hypocotyl lengths were used to fit the parameters of a mathematical model built on the known interactions of these regulators (Fig. 2).

We used four differential equations (eq. S1) that incorporated active levels of phyB, PIFs, ELF3, and COP1 and a fifth one that represented hypocotyl growth. For phyB dynamics, we adhered to the thermal reversion model previously described (*4*), where phyB is photoactivated in the light but spontaneously reversed to its inactive Pr form in the dark, whereas the rate of phyB inactivation is temperature dependent. Dynamics of ELF3 was modeled on the basis of its circadian control, with *ELF3* transcripts following a broad peak of expression at dusk (*15*, *36*). COP1 was modeled as being imported into the nucleus at a rate that varies with light and temperature, as its nuclear levels depend on interactions with several photoreceptors for which we had no experimental information (*37*).

PIF activity was modeled according to ELF3 repressing *PIF4* and *PIF5* transcription, with two additional terms incorporating phyB inhibition of PIF activity, and COP1-dependent stabilization of the PIF proteins (*30*, *32*). Last, hypocotyl growth was represented as the PIF-dependent activation of growth-related genes (*38*) and the competitive effects of ELF3, phyB, and HY5, on PIF occupancy/transcriptional activation of these genes (*1*, *7*, *15*, *25*, *28*, *39*). No additional equation was included for HY5, to which its steady-state levels were assumed to mainly depend on the proteolytic degradation by COP1 (*40*). A full mathematical definition of the model is provided in the Supplementary Materials.

**Fig. 3.**
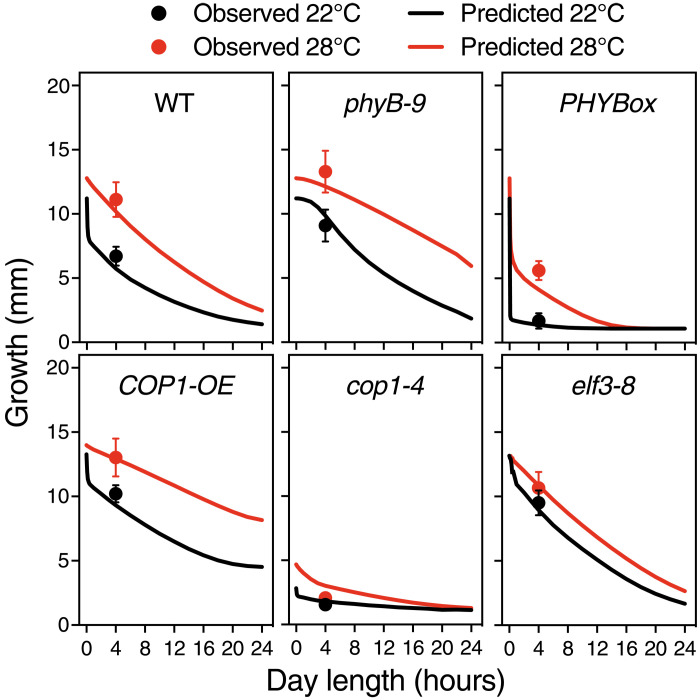
The model accurately predicts growth in a 4-hour light regime. Measured hypocotyl length (circles) for six genotypes were compared with values predicted by the model (solid lines), as indicated. Note that lines are, in this case, model predictions, not fits to the data, and hence provide a further validation of the model. Bars indicate SD of *n* = 19 to 50 seedlings (table S3).

A custom-simulated annealing algorithm (Supplementary Materials) was used to fit these different equations to the various hypocotyl growth datasets in Fig. 1A (Materials and Methods). We also used information on *ELF3* expression dynamics to constrain the fit (Materials and Methods and fig. S6). The fit showed that several parameters of the initial full model (eq. S3) were consistent with values of zero, so we proceeded to simplify the equations by eliminating these terms (eq. S1). In particular, the model worked equally well when excluding the effect of COP1 on phyB degradation and PIF action on phyB desensitization (*27*). This does not mean that these interactions do not exist but that they are quantitatively less relevant than others in our experimental conditions or that their effect is captured by other terms. Many regulatory terms affect growth in the same direction, and in consequence, our fit did not lead to a unique parameter set, but rather a high-dimensional manifold producing well-defined and consistent fits and predictions (Supplementary Materials). More experimental constraints unavailable at this moment would be necessary to narrow down dimensionality of this manifold and eventually generate a unique parameter set. The final model reproduced the dominant trends of all genetic backgrounds (Fig. 1A, solid lines), demonstrating that dynamic control of these regulators is sufficient to explain their thermomorphogenic phenotypes. Moreover, it correctly predicted the thermal responsiveness of genotypes not included in the fitting of the model (Fig. 1B) and made it possible to extrapolate their thermal behavior in other day length conditions (Fig. 3). The model predicts, for instance, that suppressed thermal responsiveness of *PHYB*ox lines is partly restored under 4-hour light. Validation of this behavior (Fig. 3) therefore shows that thermal Pfr inactivation reverses growth inhibition by excessive Pfr conversion in the light. Thermal response of double *elf3-8 phyB-9* mutants was predicted to increase linearly with day length, as seen in *phyB-9* (Fig. 1B), supporting an additive effect of ELF3 and phyB on thermomorphogenesis. In contrast, the double *phyB-9 cop1-4* mutant is nearly indistinguishable from the rest of *cop1-4* mutants, showing that enhanced COP1 action driven by Pfr thermal inactivation is key to thermomorphogenic growth (Fig. 1B). All code and data used for fitting and model simulations can be found at PabloCatalan (2022)/hypocotyl1.0 Zenodo (https://doi.org/10.5281/zenodo.6540346).

### Temperature-dependent dynamics of ELF3 and PIF4

**Fig. 4.**
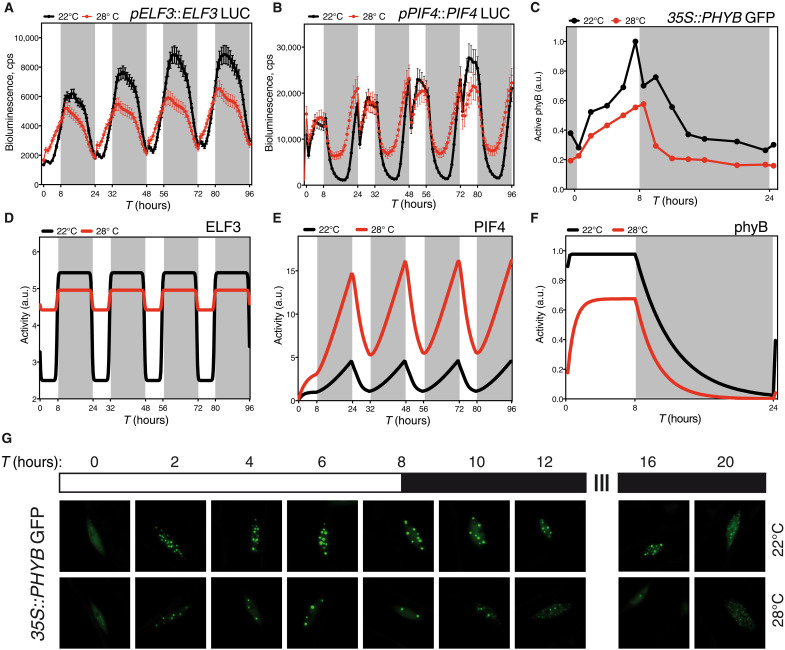
The model predicts key features of the dynamics of ELF3, PIF4, and phyB. Microplate bioluminescence detection of Col-0 lines expressing the p*ELF3::*ELF3-LUC (**A**) and p*PIF4::*PIF4-LUC (**B**) constructs. Seedlings were grown in short-day cycles at the indicated temperatures. Values represent mean ± SEM of the 2-s absolute bioluminescence of at least 24 seedlings. Plates were measured every hour. (**D**) ELF3 and (**E**) PIF4 activity as predicted by the model. (**C** and **G**) Temperature effects on phyB nuclear bodies in seedlings grown in short-day conditions. Transgenic *35S*::phyB-GFP seedlings were grown for 5 days in short-day cycles, at either 22° or 28°C. Mean size of phyB nuclear bodies and total nuclear fluorescence measured with the MATLAB software on confocal images taken every 2 hours (C) and diagram of the analyzed time points and growth conditions (G). Arbitrary units (a.u.) of phyB activity were calculated multiplying the nuclear bodies’ mean size by nuclear fluorescence at each time point. Three seedlings were measured in each assay, and two biological replicates were analyzed. All seedlings were grown under 50 μmol m^−2^ s^−1^ white light in short-day cycles at 22° or 28°C. Warmer temperature results in fewer photobodies and less nuclear intensity. (**F**) phyB activity predicted by the model. Rectangles indicate light conditions: white, lights on; gray, lights off. *T* indicates the time in hours.

ELF3 is a key node required for transmitting temperature information to the suppression of *PIF4* and *PIF5* transcription at night (*41*). We therefore used the luciferase reporter p*ELF3*::LUC, p*ELF3*::ELF3-LUC, p*PIF4*::LUC, and p*PIF4*::PIF4-LUC lines for the noninvasive imaging of temperature effects on *ELF3* and *PIF4* peak expression and protein levels. Lines expressing the p*ELF3*::ELF3-LUC (Fig. 4A) and p*PIF4*::PIF4-LUC (Fig. 4B) fusions showed identical protein profiles as p*ELF3*::ELF3-myc and p*PIF4*::PIF4-HA lines (figs. S1A and S2A, respectively), thereby allowing the analysis of rhythmic changes in these proteins during several consecutive days (Fig. 4, A and B, and Materials and Methods).

Consistent with previous reports (*15*, *36*), p*ELF3*::LUC expression showed a robust circadian rhythm peaking during nighttime (fig. S1, B and D). A roughly equivalent pattern was observed for the ELF3-LUC protein, except by showing some accumulation in short days in the light (Fig. 4A). Warm temperatures dampened p*ELF3* activity and slightly advanced its oscillation phase. Moreover, p*ELF3* waveform switched in short days to a steady-state pattern, whereby LUC activity attained a constant plateau on dark transition and returned to basal levels by end of night (fig. S1B). Reduction of ELF3-LUC activity at 28°C was lesser than for p*ELF3* transcription, indicating that ELF3 accumulates at warmer temperatures (Fig. 4A). ELF3-LUC bioluminescence was higher at 28°C during the day, whereas the protein pattern was similar at night to 22°C. The same trend is seen in Western blots conducted with p*ELF3*::ELF3-myc lines (fig. S2A), implying that temperature inactivates the EC via other molecular mechanisms than the direct control of ELF3 protein stability.

As reported in previous studies (*42*), *pPIF4*::LUC levels increased in short days shortly before dawn (fig. S2B), while in long days the promoter activity was elevated only during daytime (fig. S2D). LUC activity was then maximal in the day and quickly declined during early night. Impaired EC function at elevated temperatures advanced, in short days, p*PIF4* transcription so that expression was elevated after midnight (fig. S2B). In long days, however, *pPIF4* expression was not shifted into the night, but elevated temperatures increased the amplitude and delayed p*PIF4* oscillation phase (fig. S2, B and D).

PIF4-LUC activity followed a similar pattern as p*PIF4* expression, except for a strong stabilization of the protein in short days, at night (Fig. 4B). A similar nighttime accumulation of the protein was not observed in long days, since p*PIF4* is transcribed only in the day. Instead, PIF4-LUC levels decayed earlier than p*PIF4* expression, whereas activity of the promoter and the protein overlapped at 28°C (fig. S2C). Thus, PIF4 protein stabilization during long days late afternoon/early night may provide a window for thermal elongation, in the absence of elevated *PIF4* expression at night.

The model captures the ELF3 protein dynamics we observed experimentally (Fig. 4D and fig. S1E). Total ELF3-LUC protein levels were greater in the day at 28°C, whereas differences in the pattern of protein accumulation were reduced at night, consistent with the model’s prediction. Likewise, the model predicts PIF4 activity to be greater at night, and significantly enhanced at 28°C (Fig. 4E). PIF4-LUC protein abundance increases at 28°C during nighttime or late afternoon (Fig. 4B and fig. S2A), although it is also elevated in the day as seen in previous reports (*42*–*44*). This PIF4 pool is admitted being mostly inactive due to inhibition by phyB, HY5, or the DELLA repressors (*13*, *39*, *45*), the model thereby correctly estimating active PIF4 levels.

### Diurnal control of phyB thermal reversion

Reduced steady-state phyB Pfr levels at warmer temperatures lead to the disassembly of large phyB photobodies, thought to be active sites for phyB signaling (*5*, *46*). Formation of these subnuclear domains in bright light tightly correlates with hypocotyl growth inhibition (*47*), providing an excellent cell biology readout for phyB activity (*48*). We used *35S*::PHYB-GFP lines to monitor day length and temperature contribution to phyB nuclear dynamics. Plants were grown in short days at 22° or 28°C for 4 days, and on the fifth day, confocal images were obtained every 2 hours from the upper third portion of the hypocotyl for a 24-hour interval (Fig. 4G). The number and size of nuclear bodies were quantified in these images (fig. S3 and Materials and Methods), and active phyB levels (Fig. 4C) were extrapolated by multiplying total nuclear fluorescence (fig. S3A) by nuclear bodies’ mean size (fig. S3B). phyB-GFP fluorescence rapidly localized into discrete nuclear foci after 2-hour exposure to light (Fig. 4G), and the size of these foci gradually increased throughout the day, while their number decreased. Larger photobodies (>0.45 to 0.28 μm) subsequently declined after day-to-night transition (fig. S3, D to F), and their disassembly into smaller foci was accelerated toward the second half of the night (fig. S3, G to I). At 28°C, the number of 0.45- to 0.28-μm photobodies per nuclei was severely reduced as compared to 22°C (Fig. 4G and fig. S3, D to F). This reduction, however, was not accompanied by a greater abundance of smaller foci during the day, but only during early night (fig. S3, G to I). Quantification of total nuclear fluorescence showed that phyB abundance is substantially reduced at 28°C (fig. S3A), although relative diurnal changes in phyB-GFP fluorescence were not affected by temperature. phyB-GFP levels remained roughly constant at 22°C in the day and slowly declined at night, with an equivalent pattern observed at 28°C, except for a faster decline at night (fig. S3A).

Related temperature-dependent changes in phyB stability have been reported recently (*46*), although temperature treatments and results differed from ours. Active phyB levels steadily increased in our growth conditions in the light and reached maximal levels shortly before dusk to exponentially decay at night (Fig. 4C). phyB was also observed to be less abundant at 28°C, and large photobodies undergo a faster transition into smaller foci during early night (Fig. 4G). The model captures these dynamics, as it predicts that active phyB is reduced at warm temperatures and more rapidly inactivated in the dark (Fig. 4F).

### COP1 regulates thermomorphogenesis in the light

**Fig. 5.**
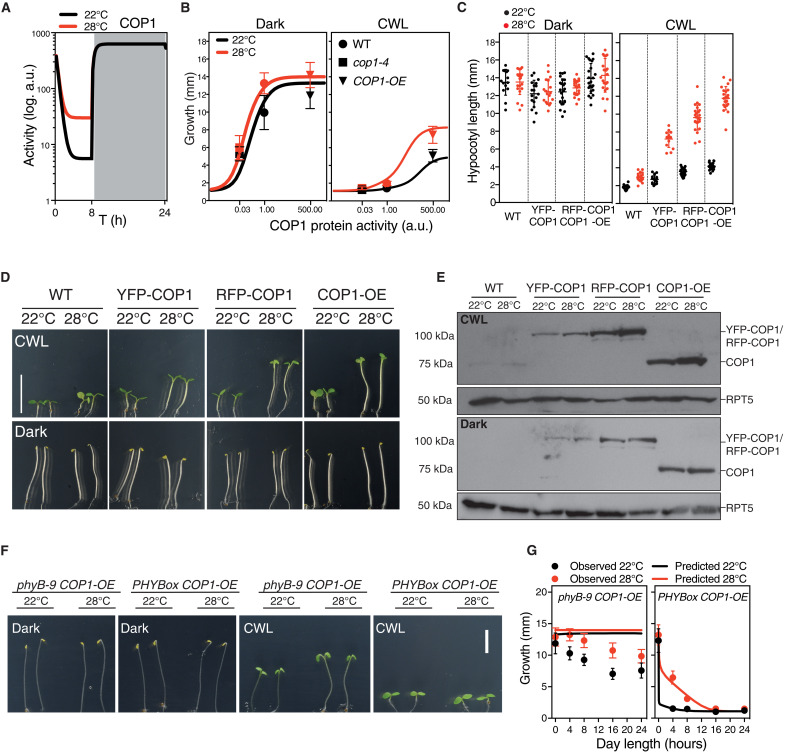
Light enhances temperature dependency of COP1 activity. (**A**) Levels of COP1 activity predicted by the mathematical model in short-day conditions. (**B**) Hypocotyl length as affected by the interaction of environmental conditions and COP1 levels. Hypocotyl lengths of Col-0, *cop1-4*, and *COP1-OE* (symbols) compared to the growth values estimated by the model (solid lines), in dark (left) and CWL (right). Bars indicate SD. (**C**) Thermal growth phenotype of different COP1 overexpressors, forming a gradient of accumulating protein levels. Bars indicate SD of *n* = 14 to 24 seedlings (table S4). (**D**) Representative pictures of 5-day-old seedlings grown in CWL (top) and continuous darkness (bottom), at 22° or 28°C. Scale bars, 10 mm. (**E**) Western blot showing that increased accumulation of the COP1 protein correlates with enhanced thermal elongation in CWL, but this response is saturated in darkness. COP1 was detected using an anti-COP1 antibody. RPT5 was used for loading control and detected using an anti-RPT5 antibody. Total protein extracts were used for immunoblot analysis. (**F**) Phenotypes of *phyB-9 COP1-OE* and *PHYBox COP1-OE* seedlings grown for 5 days in either darkness or CWL at 22° or 28°C. (**G**) Observed hypocotyl growth (circles) of *phyB-9 COP1-OE* and *PHYBox COP1-OE* lines grown for 5 days in different day length conditions, and 22° or 28°C. Solid lines are predictions from the model. Bars indicate SD of *n* = 13 to 112 seedlings (table S5).

COP1 function is essential for thermal elongation (*31*), as reflected by the severely impaired thermoresponse of *cop1-4* genotypes (Fig. 1). COP1 accumulates in the nucleus at 28°C, but higher activity in warm conditions appears to be uncoupled from light and timing information (*25*). Contrarily to these initial observations, our mathematical model predicts that COP1 activity is close to saturation and unresponsive to temperature at night, whereas its function is reduced during the day, being then critical for temperature-responsive HY5 turnover (Fig. 5A). We therefore simulated hypocotyl growth for a range of COP1 activity values and compared in Fig. 5B the weak *cop1-4* allele with the reduction in activity estimated by our fit (table S1) and the *COP1-OE* lines with the increase in activity in our fit (table S1). Running the model with these parameters captured thermal behavior of the *cop1-4* mutant and *COP1*-*OE* lines, since it predicted that reduced COP1 activity leads to impaired thermal elongation irrespective of day length, as observed for the *cop1-4* mutant or the weak *cop1-6* allele (fig. S4). Ectopic COP1 expression, by contrast, is predicted to be of no effect in darkness, but leads to taller hypocotyls and an enhanced thermoresponse in CWL, as illustrated by a remarkable increase in hypocotyl length at 28°C (Fig. 5B).

Growth of *COP1-OE* and *hy5* lines, as compared to Col-0, follows this behavior (Fig. 1A), consistently with COP1 acting at temperature signaling in the light. We thus analyzed the light-dependent thermal elongation of independent COP1 overexpressors, by using *35S*::*YFP-COP1* (*49*), *UBQ*::*RFP-COP1*, and *COP1-OE* (*50*) lines. Hypocotyl lengths of these plants were identical in darkness to the wild type, although they accumulated increasing COP1 levels (Fig. 5E), and they did not show any additional elongation at 28°C (Fig. 5, C and D). However, they displayed a thermal elongation response in CWL that was directly proportional to COP1 abundance (Fig. 5, C and E). An identical behavior was observed in continuous red (CRL) and blue light (CBL) (fig. S5A), suggesting that temperature-enhanced COP1 activity in the light is independent of phyB thermosensory function. Also, levels of the COP1 protein were slightly reduced in continuous dark (Fig. 5E), showing that saturating activity in darkness does not rely on increased COP1 abundance.

Thermal phyB Pfr inactivation is presumed to enable COP1-SPA1 re-assembly and thereby favor COP1 activity in CRL (*51*). We sought to quantify this effect by measuring hypocotyl lengths of *phyB-9 COP1-OE* and *PHYBox COP1-OE* seedlings (Fig. 5F). Modeling thermal behavior of these lines predicted an identical growth phenotype for *PHYB*ox *COP1*-OE (Fig. 5G) and *PHYB*ox plants (Fig. 1A), while *phyB-9 COP1*-OE exhibited a constitutively tall phenotype, irrespective of temperature and light conditions (Fig. 5G). *PHYB*ox *COP1*-OE lines showed, as predicted, short hypocotyls in the light and a hypersensitive response to day length thermomorphogenic inhibition as *PHYB*ox (Fig. 5G). These plants undergo thermal elongation only in short days and 4-hour light cycles, indicating that excess Pfr overrides phyB temperature reversion effects. *phyB-9 COP1-OE* lines, on the other hand, showed as predicted a tall phenotype in CWL, but retained a residual response to day length and temperature (Fig. 5, F and G). They were taller in CWL than the rest of genotypes, although elongation was partially suppressed as day length increased, presumably due to the action of other photoreceptors not considered in the model. The blue light receptors cryptochromes (CRY1 and CRY2), for instance, disrupt COP1-SPA interaction, in addition to interfering with COP1 binding to its degradation targets via a conserved VP motif (*52*–*54*). CRY1 likewise interacts in a blue light–dependent manner with PIF4, suppressing PIF4-mediated hypocotyl elongation at elevated temperatures (*55*). In line with this notion, deviation of *phyB-9 COP1-OE* growth with respect to the model is accentuated in CBL, whereas it is significantly reduced in CRL (fig. S5B). Opposing effects of red and blue light in these lines hence support a phyB-independent role of COP1 in temperature signaling, this thermal function being additively enhanced by impaired phyB activity.

### Architecture of the thermomorphogenic network

Overall, these findings unveil a specific thermal function of COP1 in the light, which was overlooked in former studies. Fitting phyB thermal inactivation parameters (*4*, *5*) to the gathered growth data enabled us to derive a minimal thermal growth model where only the essential interactions were included. The model shows that the thermal responsive network comprises a triple feed-forward coherent motif, whereby ELF3 and phyB, on one hand, and COP1, on the other, exert opposing roles on thermomorphogenic growth (Fig. 2). This network structure is typical of sensory transduction networks where the elicited response has to be fast and reversible, but it controls key physiological processes in the organism. This configuration achieves a high information transmission capacity by using an additive integration mechanism while allowing filtering out small signal fluctuations, and so it is not surprising that it was recruited during evolution to sense environmental changes in light and temperature cues (*56*).

**Fig. 6.**
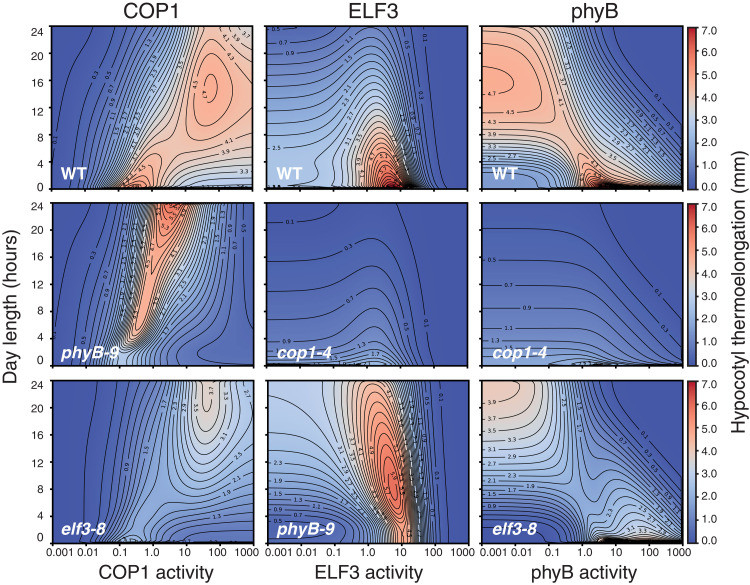
Contribution of COP1, ELF3, and phyB to day length–dependent thermoelongation as predicted by the model. Heatmap plots representing hypocotyl thermal elongation (calculated as the difference of hypocotyl length between 28° and 22°C) relative to day length (*y* axis) and activity of the COP1, phyB, and ELF3 proteins (*x* axis, as indicated). A value of 1.0 corresponds to wild-type level, while greater and lesser values are equivalent to overexpression and loss of function, respectively. Backgrounds are indicated in the panels.

Last, we used this minimal model to explore the parameter space of these coherent motifs and delimit their relative action as affected by day length duration. In Fig. 6, we show the heatmaps for the contribution of COP1, ELF3, and phyB activities (horizontal axis) to thermal elongation (growth at 28°C compared to 22°C) and how these are predicted to vary with day length (vertical axis). Also, we inferred their linked effects by generating similar heatmaps for the mutant backgrounds (as indicated in the panels). Red plot areas indicate conditions of maximal response to temperature, considering the combined effect of day length and activities of these regulators. For abscissa values = 1, moving vertically on the ordinate axes shows that thermal elongation is greater in short days (lower values in the left axis) but is significantly reduced in long days and CWL (upward in the left axis), as for our experimental data in Fig. 1. Moving horizontally along a constant day length shows impact of changes in levels of these regulators on thermal growth, i.e., for a 12-hour day length, smaller abscissa values show that reduced COP1 levels abolish thermoelongation, while thermal growth is strongly enhanced in response to increased COP1 activity (higher abscissa values), in line with the *cop1-4* and *COP1-OE* phenotypes in Fig. 1. An opposite behavior is observed for phyB activity, highlighting an opposing role of phyB and COP1 in driving thermal elongation. Thermal effects of high COP1 activity are lost in the absence of phyB, consistent with the phenotype of *phyB-9 COP1-OE* lines (Fig. 5, F and G). Furthermore, defective COP1 function impairs thermoelongation under any condition (Fig. 6, middle row, middle and right), highlighting its key contribution to thermomorphogenesis.

ELF3 is predicted to have a main thermal growth contribution in short days (Fig. 6, upper row, middle), consistent with its role in suppressing *PIF4* expression during nighttime. However, phyB is found to override ELF3 activity, as reflected by the enhanced thermal growth, over a wider range of day lengths, caused by elevated ELF3 in the *phyB-9* background (Fig. 6, lower row, middle). Unexpectedly, a small elevation in ELF3 is predicted to favor thermal elongation, whereas greater ELF3 levels reverse this effect (Fig. 6). LUX was actually shown to bind with high affinity the LBS recognition motif, whereas its activity was abolished on dimerization with ELF3 and restored on additional interaction with ELF4 (*8*). It is thus conceivable that ELF3 counteracts LUX inhibition of *PIF4* and its own expression, until LUX elevation and higher EC levels reverse this positive effect. Although this dual function remains to be substantiated, it is noteworthy that it is predicted to be potentiated by loss of phyB activity, hence underscoring a role of phyB signaling in antagonizing ELF3. Notably, COP1 thermal activity appears also in these simulations to be affected by the lack of ELF3, suggesting that the ELF3 and COP1 function is somehow linked (Fig. 6, lower left). Thus, it will be interesting in future studies to validate these novel interactions and investigate their underlying molecular mechanisms.

## DISCUSSION

Thermal responsiveness is pivotal in nature to reduce the impact of potentially damaging temperatures. The magnitude of this response is, however, reduced on exposure to full sunlight and longer day lengths of summer, which often overlap with warmer conditions. Understanding this inhibition at the molecular level is thus essential to optimize resilience to climate change of long day–requiring crops.

In this work, we showed that COP1 acts as a major thermal regulator during daytime. COP1 accumulates in the nucleus at elevated temperatures (*25*), and its activity is essential for thermormophogenic elongation. Thermal elongation in CWL is directly correlated with COP1 protein levels, suggesting that warm temperatures interfere with COP1 nuclear exclusion in the light. COP1 targets multiple transcriptional factors antagonizing PIF activity for degradation, besides mediating ELF3 destabilization (*26*, *28*). COP1 function is also required for up-regulated *PIF4* expression and protein accumulation at warm temperatures (*25*, *57*), and thus, it is conceivable that its role in thermal signaling involves a combination of these processes.

Direct phyB and SPA1 interaction is established to interfere with COP1 activity (*51*). Hence, it cannot be excluded that light-dependent COP1 thermal function is primarily a consequence of phyB Pfr thermal inactivation. Our results, however, do not support this mechanistic model. Loss of phyB or ELF3 function in the double *phyB-9 cop1-4* and *elf3-8 cop1-4* mutants is unable to rescue thermal elongation defects of the *cop1-4* allele, revealing that COP1 activity is crucial for the enhanced thermal responsiveness of the *phyB-9* and *elf3-8* backgrounds (Fig. 1). COP1 promotion of thermal growth in CWL is additive to the *phyB-9* mutation, suggesting that thermal COP1 function is to an important extent independent of phyB activity. While *phyB-9 COP1*-*OE* lines were not as elongated in CWL as predicted by the model (Fig. 5G), this divergence was notably reduced in CRL, while it was aggravated in CBL (fig. S5B). This supports that this phenotype is caused by direct COP1 inhibition by the blue light receptor cryptochromes (*58*, *59*), calling for future improvement of the model on incorporating these photoreceptors.

Thermoelongation heatmaps (Fig. 6) predict that phyB and COP1 activities are more relevant as day length increases, while the role of ELF3 is more prominent in short days. Both lower phyB and higher COP1 levels lead to significantly enhanced thermal elongation in long days and CWL, while defective COP1 function abolishes thermal responsiveness irrespective of phyB and ELF3 levels. Therefore, the model fully captures the epistatic effects of *cop1-4* on loss of phyB or ELF3 function, supporting a pivotal role of COP1/HY5 in modulating PIF4 activity downstream of these regulators. Heatmap simulations also show that impaired ELF3 activity reduces phyB and COP1 thermal effects, consistent with defective EC function leading to a de-repressed response at 22°C.

Diurnal ELF3 oscillation was coarsely introduced when fitting the model, while PIF4 activity was left unconstrained. In its current configuration, the model correctly predicts that PIF4 activity is enhanced at elevated temperatures, and this effect is greater in short days, but it is unable to predict diurnal timing of this regulation. Thus, upgrading the model with the LUC dynamics of these proteins will certainly improve its already strong predictive power.

Most crop species show a narrow genetic variability in thermomorphogenic-related traits (*60*), which threatens their productivity in a global warming scenario. Natural variation in the *phyB* and *ELF3* loci was found to be associated with altered thermal sensitivity in *Arabidopsis* and several crops, in a consistent manner with their adaptation to local climate variables (*6*, *9*, *61*, *62*). Lower activity of these alleles results in an elongated phenotype that is also associated with earlier flowering and reduced yield. Here, we showed that COP1 activity is essential for increased thermal responsiveness of *phyB-9* and *elf3-8* mutants and that COP1 overexpression leads to enhanced thermomorphogenic growth in the light but a wild-type response in darkness. *COP1-OE* lines, on the other hand, do not exhibit the same accelerated flowering as *phyB-9* and *elf3-8* mutants. Hence, allelic variants in the *COP1* gene that lead to an overall increase in protein levels or activity emerge from this work as excellent candidates for optimizing crops’ resilience to climate change.

## MATERIALS AND METHODS

### Plant materials

*Arabidopsis thaliana* Col-0 plants were used as wild type in this study. The mutants and transgenic lines used in the different experiments were as follows: *elf3-8* (*63*), *ELF3*ox (*26*), *pif4-101* (*64*), *pifq* (*65*), *phyB-9* (*66*), *COP1*ox (*26*), *COP1-OE* (*50*), *cop1-4* (*67*), *cop1-6* (*67*), *hy5-215* (*68*), *elf3-8 cop1-4* (*26*), *ELF3*ox *cop1-4* (*26*), *PIF4*-HA (*69*), *pELF3::ELF3*-myc (*7*), *pPIF4::PIF4*-HA (*43*), *cop1-4*/*35S:YFPCOP1* (*49*), and *35S:PHYB-GFP* (*70*).

### Growth conditions

Seeds were surface-sterilized for 15 min in 70% (v/v) ethanol and 0.1% (v/v) Tween 20, followed by two washes of 2 min in 96% (v/v) ethanol. Seeds were air-dried and sown on half-strength MS agar plates with 1% sucrose and stratified for 3 days at 4°C in the dark. Germination was synchronized by illuminating the plates with white light for 4 hours and transferring them back to darkness for 20 additional hours. Seedlings were then grown under specific temperature and day length conditions with white light (50 μmol m^−2^ s^−1^), red light (35 μmol m^−2^ s^−1^), or blue light (35 μmol m^−2^ s^−1^).

### Plasmid constructs and generation of *Arabidopsis* transgenic lines

To generate the p*ELF3*::LUC expression cassette, a fragment containing the 2.21-kb upstream regulatory region of ELF3 was amplified from Col-0 genomic DNA using primers ELF3Prom-Fw (5′-CACCCTTATAAATAAAATTCC-3′) and ELF3Prom-Rv (5′-CACTCACAATTCACAACCTTTTTC-3′) and cloned into the Gateway pENTRY vector (pENTR Directional TOPO Cloning kit, Invitrogen) to obtain the pELF3-TOPO plasmid. To generate the *pELF3::ELF3*-LUC construct, the ELF3 coding region was amplified without the stop codon using the ELF3 cloning-Fw/ELF3 cloning-Rv primers and then cloned into pENTR/D-TOPO vector. The pELF3 sequence was amplified from *pELF3-TOPO* with ELF3 Prom NotI-Fw/ELF3 Prom NotI-Rv and then inserted into Not I of ELF3-TOPO to produce the p*ELF3::ELF3*-TOPO plasmid. Last, *pELF3-TOPO* and p*ELF3::ELF3*-TOPO constructs were mobilized by LR clonase II recombination (Invitrogen) into the pLUC-Trap3 vector (*71*).

To generate the p*PIF4*::LUC reporter gene, we amplified a 2.47-kb fragment upstream the PIF4 ATG with primers PIF4Prom-Fw (5′-CACCCAGTACGCATCCAATCTTCTC-3′) and PIF4Prom-Rv (5′-CGGGATCCGGGTACAGACAGAAAGTGAC-3′) and followed the same procedure as described above for p*ELF3*::LUC. The *pPIF4*::PIF4-TOPO construct (*43*) was transferred to the pLUC-Trap3 destination vector.

*COP1* CDS was amplified using primers COP1cloning-Fw (5′-CACCATGGAAGAGATTTCGACGGATC-3′) and COP1cloning-Rv (5′-GAAGATCTTCACGCAGCGAGTACCAGAAC-3′) and cloned into the pENTR/D-TOPO vector. The binary vector pUBN-RFP-Dest (*72*) was used for N-terminal fusion of the mRFP tag.

To generate *Arabidopsis* transgenic lines, the *Agrobacterium*-mediated floral dip method was used to transform p*ELF3*-LUC, p*ELF3*::*ELF3*-LUC, p*PIF4*-LUC, and p*PIF4::PIF4*-LUC plasmids into Col-0, whereas *pUBN-RFP-COP1* was transformed into the *cop1-4* mutant background. Homozygous T3 plants were used in this study.

The double homozygous *phyB-9 COP1-OE* and *PHYBox COP1-OE* were generated by crossing the transgenic *COP1-OE* line with *phyB-9* and *PHYBox*, respectively. The *phyB-9* mutation was genotyped using primers phyB-9-Fw (5′-GCAATGCCACACCTGTTCTTGTGG-3′) and phyB-9-Rv (5′-CTTCACTAGGAGCAACACCCAACG-3′). phyB and COP1 overexpression constructs were polymerase chain reaction (PCR)–genotyped with primers 35S-fw (5′-CCACTGACGTAAGGGATGAC-3′) and phyB-9-rv and COP1-PE-fw (5′-CTTCCCTCCGTACTACACTCTTATC-3′), respectively.

### Hypocotyl measurements

Seeds were sown on half-strength MS agar plates with 1% sucrose and stratified for 4 days at 4°C in darkness. After stratification, the germination was induced by placing the plates for 4 hours under 50 μmol m^−2^ s^−1^ white light and 20-hour darkness at 22°C. After 5 days at the indicated photoperiod and temperature regimes, seedlings were photographed and hypocotyl length was determined using ImageJ software.

### Luciferase activity assays

Luciferase activity assays were carried out using the LB 960 Microplate Luminometer (Berthold Technologies, UK). Transgenic *Arabidopsis* seedlings were plated on 0.5× MS agar medium, and after 1 to 2 days of germination, they were transferred to 96-well microplates (Costar, USA) filled with 175 μl of 0.5× MS agar medium 1% sucrose and 35 μl (50 μg/ml) of Beetle Luciferin (Promega) dissolved at 10 mg/ml in dimethyl sulfoxide. Levels of luciferase activity were registered every hour and represented as the average of counts per 2 s in each well, with at least 24 plants per line. The luminometer was housed in a growth chamber to maintain the plants under controlled growth conditions.

### Confocal microscopy

Confocal fluorescence images of phyB-GFP were taken from the epidermis and the first subepidermal layers of the upper third portion of the hypocotyl. We used an LSM5 Pascal laser scanning microscope (Zeiss) with a water-immersion objective lens (C-Apochromat 40×/1.2; Zeiss). Probes were excited with an argon laser (λ = 488 nm), and fluorescence was detected using a BP 505-530 filter. Images were taken from individual nuclei, and image analysis was performed as in (*5*). Each data point consists of two replicates coming from two independent experiments showing the same trend. Replicates consisted of the average of three plants coming from the same plate, and three nuclei per plant were analyzed.

### Western blot analysis

Total protein extracts from an equal number of *Arabidopsis* seedlings were prepared by homogenizing plant material in extraction buffer containing 125 mM tris-HCl (pH 7.4), 2% SDS, 10% glycerol, 6 M urea, and 1% β-mercaptoethanol. The extracts were heated at 95°C for 3 min and then centrifuged at 4°C for 15 min to recover the supernatant. Protein samples were boiled for 5 min in TMx2 loading buffer, and 40 μl of the protein extracts was run on 8% SDS–polyacrylamide gel electrophoresis gels. Homogeneous protein transfer to nitrocellulose membranes (Whatman) was confirmed by Ponceau red staining. For PIF4 and ELF3 detection, blots were respectively immunodetected with an anti-hemagglutinin (HA) peroxidase (Roche) or anti-myc antibody (Abmart), followed by incubation with anti-mouse horseradish peroxidase (HRP)–conjugated antibody. Anti-RPT5 (Enzo) was used for equal loading control. For COP1 immunodetection, it was used as an anti-COP1 antibody supplied by U. Hoecker (*73*), followed by incubation with an HRP-conjugated anti-rabbit secondary antibody. Chemiluminescence was detected with the SuperSignal West Pico and Femto substrates (Pierce).

### Model fit

The mathematical model was fitted to the data in Fig. 1A and fig. S6 using a simulated annealing algorithm (*74*). The data are not sufficient to constrain the fit to a unique parameter set, but the model defines a manifold that makes consistent and well-defined predictions independently of the parameter set (see the Supplementary Materials for details).

## Supplementary Materials

### This PDF file includes:

Figs. S1 to S16 Supplementary Text Tables S1 to S6 References
