# Supplementary material for: COP1 dynamics integrate conflicting seasonal light and thermal cues in the control of Arabidopsis elongation
Source: Sci Adv. 2022 Aug 19;8(33):eabp8412. doi: 10.1126/sciadv.abp8412 (PMC9390991; doi:10.1126/sciadv.abp8412)
Supplement: Supplementary file 1 — Figs. S1 to S16 Supplementary Text Tables S1 to S6 References [file sciadv.abp8412_sm.pdf]

Science Advances
AAAS

Supplementary Materials for

COP1 dynamics integrate conflicting seasonal light and thermal cues in the control of *Arabidopsis* elongation

Cristina Nieto *et al.*

Corresponding author: Saúl Ares, sares@cnb.csic.es; Salomé Prat, salome.prat@cragenomica.es

Sci. Adv. **8**, eabp8412 (2022)
DOI: 10.1126/sciadv.abp8412

This PDF file includes:

Figs. S1 to S16
Supplementary Text
Tables S1 to S6
References

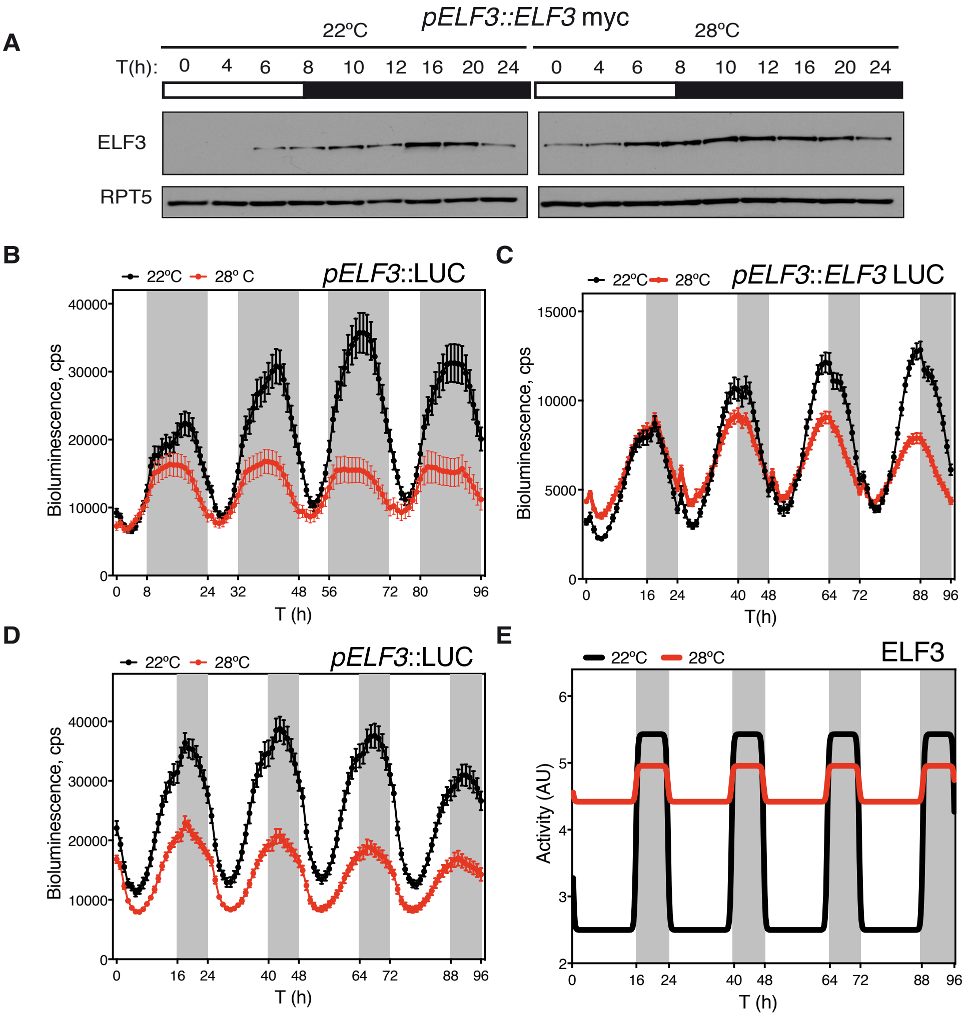

Fig. S1. Effect of warm temperature on ELF3 expression and protein abundance.
(A) Total protein extracts from *pELF3*::ELF3-myc transgenic lines were analyzed by Western blot using an anti-myc antibody. RPT5 was used for loading control and detected using an anti-RPT5 antibody. Seedlings were grown for 5 days at 22ºC or 28ºC under short day conditions. Samples were collected at the indicated time points. **(B)** Bioluminescence detection of Col-0 lines expressing the *pELF3*::LUC construct, grown in short days. **(C and D)** Bioluminescence recorded from transgenic seedlings expressing the *pELF3*::ELF3-LUC and *pELF3*::LUC constructs, grown in long days and 22ºC/28ºC. Values represent mean ± SE of the 2 sec absolute bioluminescence of at least 24 seedlings. **(E)** ELF3 activity predicted by the model in long days.

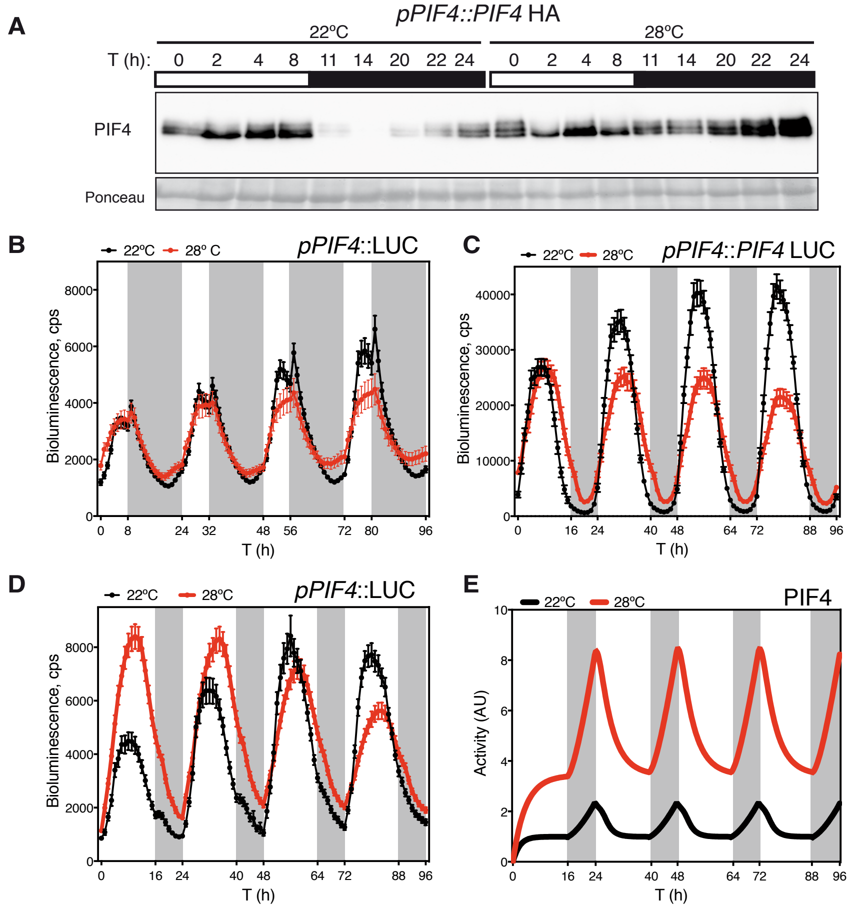

Fig S2. Effect of warm temperature on PIF4 expression and protein abundance.
(A) Western blot of *pPIF4*::*PIF4*-HA transgenic lines. The PIF4-HA protein was detected using an anti-HA anti-body. Ponceau staining was used as loading control. Rectangles indicate light conditions: white, lights on, and grey, lights off. Samples were collected at the indicated time points. **(B)** Bioluminescence detection of Col-0 lines expressing *pPIF4*::LUC construct, grown in short days. **(C and D)** Bioluminescence recorded from transgenic seedlings expressing the *pPIF4*::*PIF4*-LUC and *pPIF4*::LUC constructs, grown in long days and 22ºC/28ºC. Values represent mean ± SE of 2 sec absolute bioluminescence of at least 24 seedlings. **(E)** PIF4 activity predicted by the model in long days.

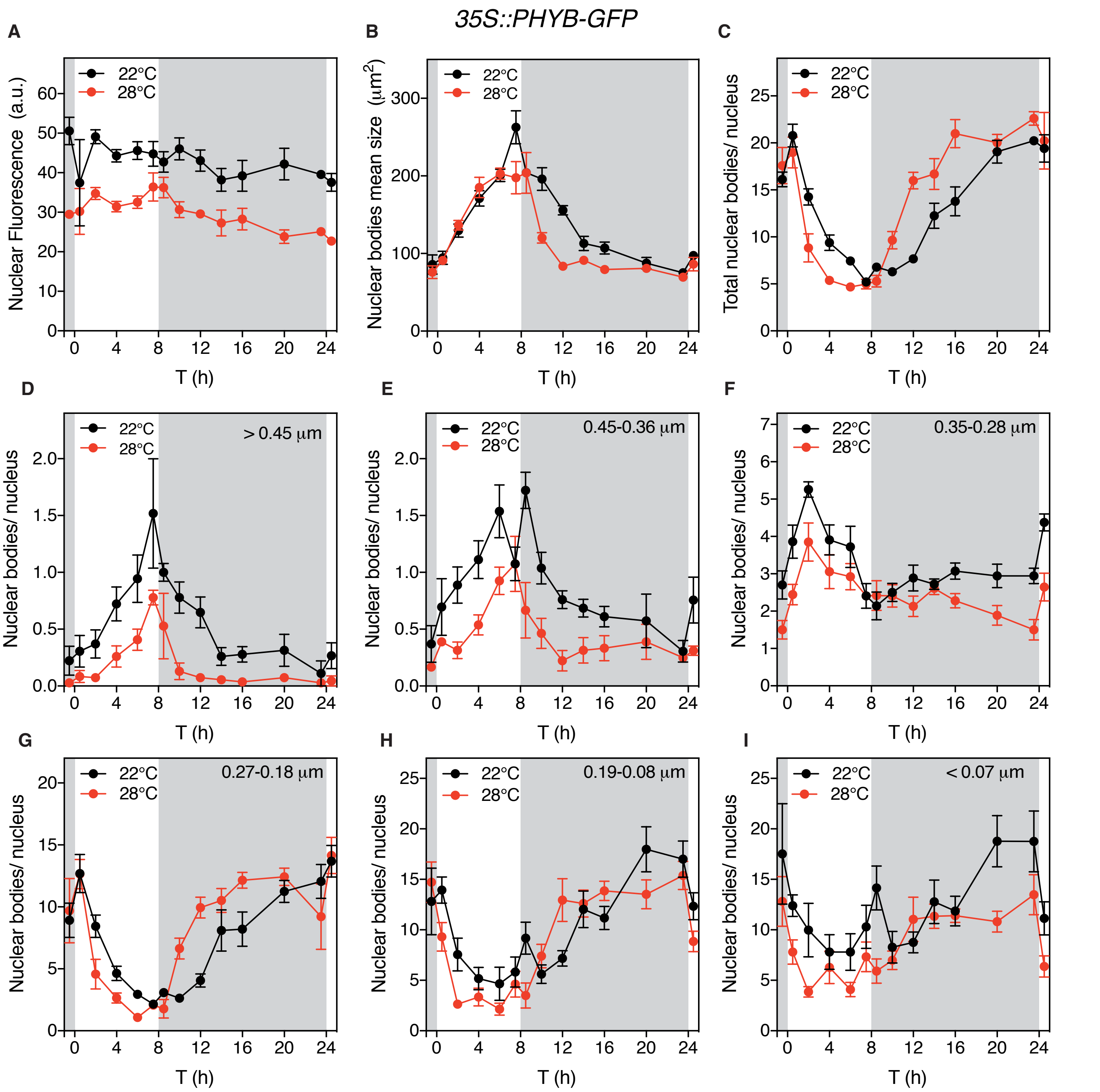

Fig. S3. phyB nuclear bodies formation is affected by temperature.
35S::PHYB-GFP transgenic seedlings were grown for 5 days under 50 µmol.m^{-2}.s^{-1} white light in short day cycles and 22ºC or 28ºC. **(A)** Total nuclear fluorescence expressed in arbitrary units (a.u.). **(B)** Nuclear photobodies mean size (µm^2). **(C)** Number of total bodies per nucleus. **(D-I)** Number of phyB nuclear bodies, sorted by size categories, as measured with Matlab software. The rectangles indicate the light conditions: white, lights on, and grey, lights off. Values represent mean ± SE of two independent experiments. Each replicate is the average of 3 seedlings and 3 nuclei/plant were analyzed. T(h) indicates time in hours.

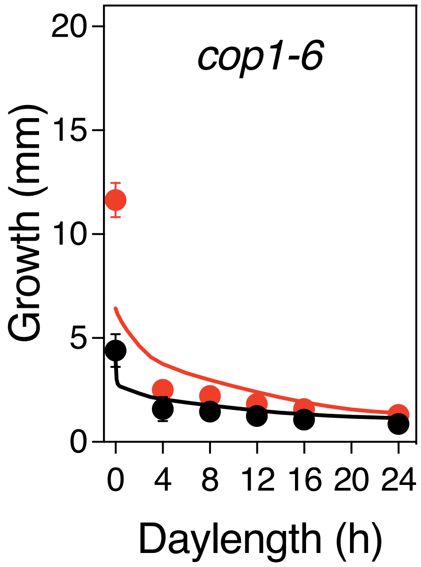

Fig. S4. Impaired temperature-induced elongation of the weak *cop1-6* allele.
Seedlings were germinated at 22ºC and grown at either 22ºC or 28ºC and darkness, short days (8h light), 12h light, long days (16h light) and continuous white light. Plates were incubated for 5 days under these conditions and photographed for hypocotyl length measurements with ImageJ. Circles represent mean ± SD, number of seedlings indicated in table S2. Solid lines show the growth fitted by the model as a reduction of COP1 activity. Note that *cop1-6* is described as a temperature-conditional mutation (*75*). The *cop1-6* allele carries a G to A mutation at the intron 4 acceptor splicing junction that produces a full-length protein with a 5 amino acid insertion in the NLS. This insertion interferes with nuclear accumulation of the cop1-6 protein at low temperatures, but not in darkness and 28ºC (*76*). The *cop1-6* allele thus shows an identical thermal behavior as *cop1-4* in all growth conditions except in darkness and 28ºC, where it shows a wild type phenotype. This behavior is not described in the model, and for this reason it fails to reproduce it.

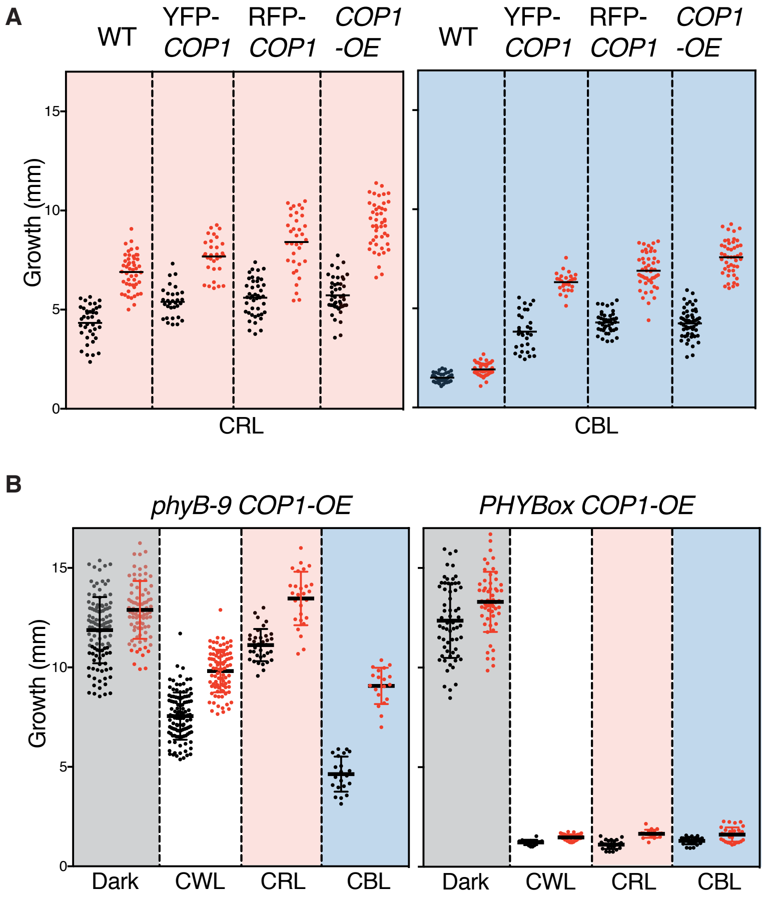

Fig. S5. Thermal response of COP1 lines in red and blue light.
(A) Hypocotyl length phenotypes of the different COP1 overexpressor lines in continuous red (CRL) and continuous blue (CBL) light, n=27-57 seedlings (table S6). **(B)** Phenotypes of *phyB-9 COP1-OE* and *PHYBox COP1-OE* seedlings grown for 5 days either in darkness, CWL, CRL and CBL, at 22ºC or 28ºC. Bars indicate standard deviation of n=13-112 seedlings (table S5).

COP1 dynamics integrate conflicting seasonal light and thermal cues in the control of *Arabidopsis* elongation

Supplementary Text to: Nieto et al., *Sci. Adv.* **8**, eabp8412 (2022),

DOI: 10.1126/sciadv.abp8412

Cristina Nieto[1,2†], Pablo Catalán[3,4†], Luis Miguel Luengo[1,5],
Martina Legris[6], Vadir López-Salmerón[1], Jean Michel Davière[1],
Jorge J. Casal[6,7], Saúl Ares[1,3*] and Salomé Prat[1,5*]

[1]Centro Nacional de Biotecnologia (CNB), CSIC, Darwin 3, 28049 Madrid, Spain

[2]Centro de Recursos Fitogeneticos y Agricultura Sostenible (CRF-INIA), CSIC, Autovia A2, km 32, 28805 Alcala de Henares, Madrid, Spain

[3]Grupo Interdisciplinar de Sistemas Complejos (GISC), Madrid, Spain

[4]Department of Mathematics, Universidad Carlos III de Madrid, Avenida de la Universidad 30, 28911 Leganes, Madrid, Spain

[5]Centro de Investigación en Agrigenomica (CRAG), CSIC-IRTA-UAB-UB, 08193 Cerdanyola, Barcelona, Spain

[6]Fundación Instituto Leloir, Instituto de Investigaciones Bioquímicas de Buenos Aires, Consejo Nacional de Investigaciones Científicas y Técnicas, 1405 Buenos Aires, Argentina

[7]Instituto de Investigaciones Fisiológicas y Ecológicas Vinculadas a la Agricultura, Facultad de Agronomía, Universidad de Buenos Aires, Consejo Nacional de Investigaciones Científicas y Técnicas, 1417 Buenos Aires, Argentina

[*]Corresponding author. Email: sares@cnb.csic.es (S.A.); salome.prat@cragenomica.es (S.P.)

[†]These authors contributed equally to these work

Code availability

All code and data to fit and simulate the mathematical model can be found at:

Pablo Catalán. (2022). PabloCatalan/hypocotyl: hypocotyl1.0 (hypocotyl). Zenodo.

https://doi.org/10.5281/zenodo.6540346

Mathematical model

Final model

The mathematical model used to obtain the results in the main text is as follows:

$$\begin{aligned}
\frac{dB(t)}{dt} &= p_B(T)L(t)(mut_B - B(t)) - k_r(T)B(t) \\
\frac{dE(t)}{dt} &= p_E(t,T,D,mut_E) - d_E E(t) \\
\frac{dC(t)}{dt} &= mut_C\left[p_{CL}(T)L(t) + p_{CD}(1-L(t))\right] - d_C C(t) \\
\frac{dP(t)}{dt} &= mut_P \frac{p_P}{1+p_{PE}(T)E(t)} - \frac{d_P}{1+k_{PC}C(t)}P(t) - d_{PB}B(t)P(t) \\
\frac{dG(t)}{dt} &= p_G + k_G \frac{p_{GP}P(t)}{1+p_{GP}P(t)+p_{GE}E(t)+p_{GB}B(t)+\frac{p_{GH}}{1+p_{HC}C(t)}},
\end{aligned} \tag{1}$$

where t is time, $B(t)$ represents the concentration of the active form of phyB, Pfr, in the nucleus; $E(t)$ represents the concentration of ELF3 protein in the nucleus; $P(t)$ represents the nuclear concentration of PIF1, PIF3, PIF4 and PIF5; $C(t)$ represents COP1 in the nucleus, and $G(t)$ measures hypocotyl growth in mm. $G(t)$ is an effective variable used as proxy for the expression of PIF-targeted, growth related genes. Parameters that are a function of T (temperature) can have different values at 22°C and 28°C. $L(t)$ represents light, and it is either 0 at night, or 1 during daytime.

Parameters have the following meaning: for a given species K, d_K is the decay rate of molecule K, p_B is phyB's rate of activation and traslocation to the nucleus under light; k_r is the rate of dark reversion, which following earlier reports (4,5) we assume happens during the day and night; p_P is PIFs' production rate; p_{PE} is the intensity of ELF3's inhibition of *PIFs* expression; k_{PC} is the intensity of COP1's inhibition of PIFs degradation; d_{PB} is the intensity of phyB's promotion of PIFs degradation and inactivation; p_{CL} and p_{CD} are, respectively, COP1's production rates during the day and the night; p_G is the basal rate of hypocotyl growth; k_G is the conversion between PIFs targets gene expression and growth; p_{GK} is molecule K's intensity of its effect on growth, p_{GH} is related to the levels of HY5 (see below), and p_{HC} is the intensity of COP1's inhibtion (through degradation) of HY5. Finally, mut_K is a multiplier that alters molecule K's production to acommodate knock-out and over-expressor lines; i.e. $mut_K = 1$ for the wild-type, $mut_K < 1$ for weak mutants and $mut_K > 1$ for over-expressor lines. In phyB and ELF3's case, knock-out mutants $phyB$ and $elf3.8$ have $B = 0$ and $E = 0$ at all times, respectively;

the mut_E parameter in Eq. (2) below is only used with values greater than 1 to model over-expression.

ELF3's expression follows a quasi-square wave:

$$p_E(t,T) = \begin{cases} mut_E\, p_{E1}(T) + p_{E2}(T) & \text{if } D = 0 \text{ hours.} \\ mut_E\, p_{E1}(T) - p_{E2}(T)\left(-1 + \frac{2}{1+\exp(-k_0 t_0)} - \frac{2}{1+\exp(-k_0 t_1)} + \frac{2}{1+\exp(-k_0 t_2)}\right) & \text{if } 0 < D < 24 \text{ hours.} \\ mut_E\, p_{E1}(T) - p_{E2}(T) & \text{if } D = 24 \text{ hours.} \end{cases} \quad (2)$$

where D is the number of hours of light in the day; $p_{E1} + p_{E2}$ and $p_{E1} - p_{E2}$ are ELF3's average production in darkness and light, respectively; $t_0 = t \mod 24$, $t_1 = t_0 - D$ and $t_2 = t_0 - 24$, and $k_0 = 5$ h^{-1}. With this function, ELF3 oscillates between $p_{E1} + p_{E2}$ and $p_{E1} - p_{E2}$ rapidly. The advantage over using a simpler square wave is that this function is smooth, which prevents numerical anomalies. The value $k_0 = 5$ h^{-1} defines the timescale of the rise and fall of the function when changing light conditions. It has been assigned arbitrarily to produce a smooth function but maintaining a sharp distinction between expression during light and darkness. Our results do not depend on the exact value, within reason, of this parameter.

We also assume that the over-expressor line $ELF3ox$ increases ELF3's production level p_{E1}, but that day-night oscillations, represented by p_{E2}, are maintained.

Development of the model

We have used the following experimental observations in order to develop the initial model:

1. phyB is activated by light and tends to spontaneously revert back to its inactive form. This 'dark reversion' is faster with higher temperatures. We follow the modeling in Jung et al. (4) of phyB's activation and dark reversion with small modifications.

2. phyB is marked for degradation by COP1, and this degradation constant is enhanced by PIFs (27).

3. phyB mediates the degradation of PIF4 and PIFs enhance the degradation of phyB (29,30).

4. ELF3 is transcribed less during the day, and more during the night, in a sinusoidal pattern (15).

5. phyB physically interacts with ELF3, and this could potentially stabilize ELF3 (28).

6. COP1 also marks ELF3 for degradation (26).

7. PIF4 expression is suppressed by ELF3 as part of the evening complex (15).This regulation is weaker at warmer temperatures, as the EC is impaired by temperature (6).

8. COP1 stabilizes PIF4 and PIF5 (32,33).

9. COP1 is inactivated by phyB (19).

10. Hypocotyl growth is enhanced by the PIFs (10).

11. phyB prevents PIFs from binding to their targets (12,13).

12. ELF3 (independently from the evening complex) also prevents PIFs from binding to their targets (7,28).

13. HY5 represses hypocotyl growth, while COP1 mediates degradation of HY5 (16).

From these interactions, we developed the following model:

$$
\begin{aligned}
\frac{dB(t)}{dt} &= p_B(T)L(t)(mut_B - B(t)) - k_r(T)B(t) - d_{BC}C(t)B(t) - d_{BP}P(t)B(t)\\
\frac{dE(t)}{dt} &= p_E(t,T,D,mut_E) - d_{EC}C(t)E(t) - \frac{d_E}{1+k_{EB}B(t)}E(t)\\
\frac{dC(t)}{dt} &= mut_C\left[p_{CL}(T)L(t) + p_{CD}(T)(1-L(t))\right] - d_C C(t) - d_{CB}B(t)C(t)\\
\frac{dP(t)}{dt} &= mut_P\frac{p_P}{1+p_{PE}(T)E(t)} - \frac{d_P}{1+k_{PC}C(t)}P(t) - d_{PB}B(t)P(t)\\
\frac{dG(t)}{dt} &= p_G + k_G\frac{p_{GP}P(t)}{1+p_{GP}P(t)+p_{GE}E(t)+p_{GB}B(t)+p_{GH}H(t)}.
\end{aligned}
\tag{3}
$$

Here $H(t)$ is HY5 concentration. As we do not have an equation for HY5, we assume it is in equilibrium and that its average levels are determined only by COP1: $H(t) = p_H/(1+p_{HC}C(t))$. The parameter p_H is therefore no longer necessary and the effect of varying it is equivalent to variation of p_{GH}. Due to lack of additional information, we set the initial conditions for all variables to zero for our numerical simulations. That is, $B(0) = P(0) = E(0) = C(0) = G(0) = 0$.

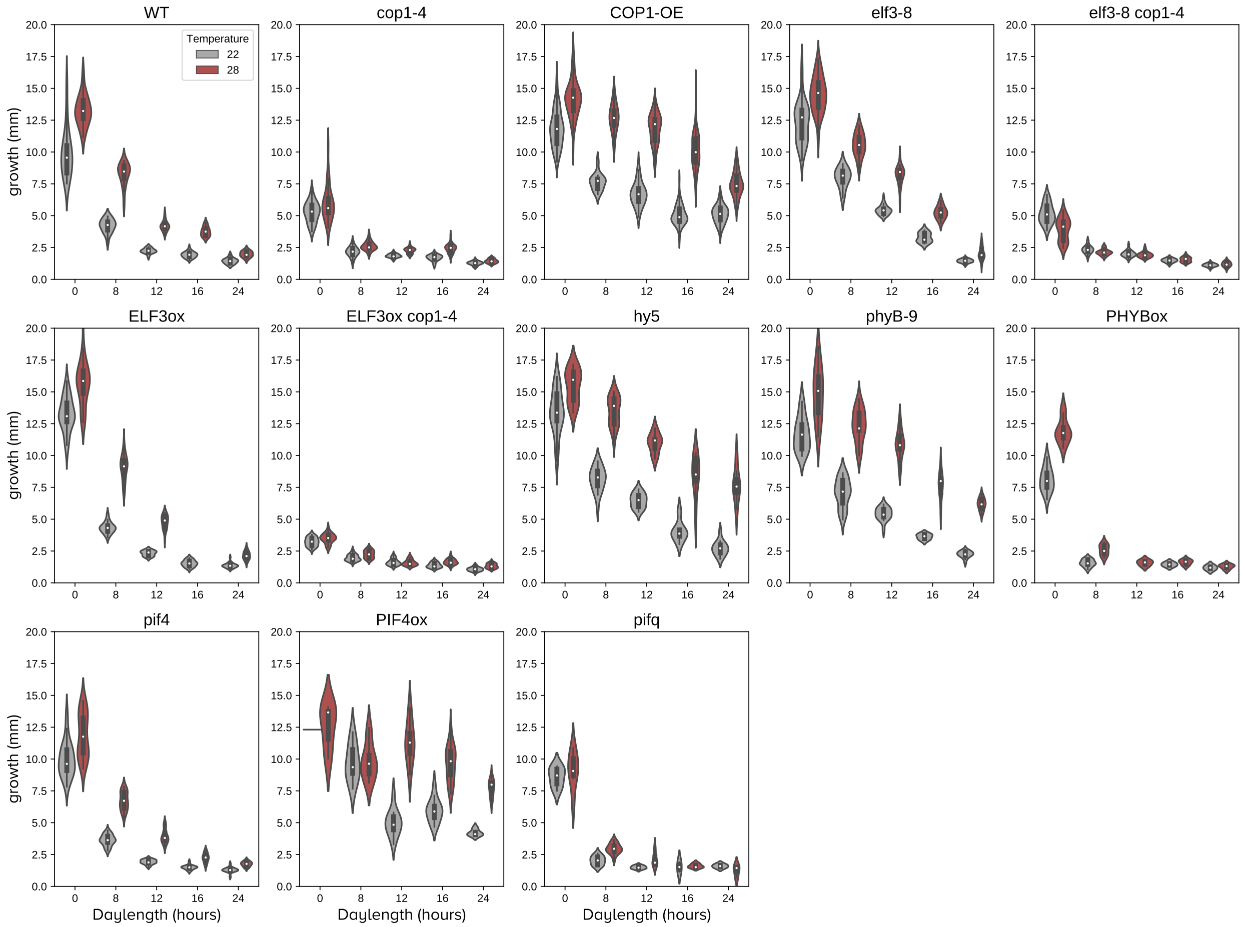

Figure S6: **Experimental data used to fit the model (I).** We grew several mutant and over-expressor lines under different temperature and photoperiod conditions for five days (Methods), and used final hypocotyl length to fit our mathematical model. This figure is a replot of Fig. 1A, using a violin plot that shows the distribution of all the individual values resulting in each data point.

Simulated annealing

We wrote a custom simulated annealing algorithm (74) to fit Eqs. (3) to our experimental data (Fig. 1A, main text). We simulated the model for 5 days under all experimental conditions and for all genotypes and tried to minimize an energy function that was the sum of all the squared errors between our experimental data, fig. S6, and the model predictions. Together with growth data, we also used differences between model predictions and ELF3 levels in Col-0 and *phyB-9* backgrounds, fig. S7. The energy function was

$$E = \sum_{k\in\mathcal{G}} (o_k - e_k)^2 + w \sum_{k\in\mathcal{E}} (o_k - e_k)^2 \tag{4}$$

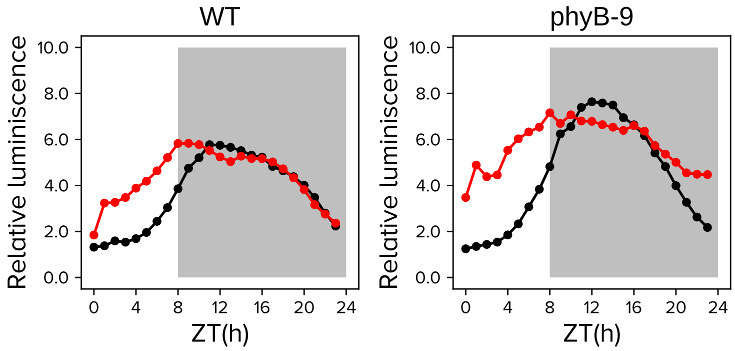

Figure S7: **Experimental data used to fit the model (II).** We measured ELF3 levels using *pELF3*::ELF3 LUC transgenic lines under two different backgrounds: Col-0 and *phyB-9*, and used that information to fit our model. Data from a similar experiment in the Col-0 background is also plotted in Fig. 4A.

where $\mathcal{G}$ and $\mathcal{E}$ are the sets of growth and expression datapoints, respectively, and w is the weight we give to expression data. We used $w = 1$, that is, each datum contributed equally to the function.

We used $3,544$ growth datapoints and 96 expression datapoints (see the Github repository for more details). We fixed $k_r(22) = 0.232$ h^{-1} and $k_r(28) = 0.411$ h^{-1}, based on experimental measurements from Jorge Casal's lab, see Ref. (5); $p_B(22) = 10$ in order to follow Ref. (4); furthermore, p_P and $p_{CL}(22)$ were set to 1 in order to reduce the dimensionality of the search space. All other parameters were set to 1 at the beginning of the search, allowing them to change ($p_{E2}(22)$ and $p_{E2}(28)$ were set to 0.9 at the beginning of the search so as to allow variation in ELF3's production).

We started the process with all parameters set to 1. At each step i, we perturbed a randomly chosen parameter by adding to it a Gaussian random number with mean 0 and variance 0.1, always checking that no parameter became negative. For this perturbed set of parameters, we computed the energy function E_{new}, compared it with the energy of the old parameter set E_{old}, and accepted the set with probability

$$p_{\text{acc}} = \begin{cases} 1 & \text{if } E_{\text{old}}/E_{\text{new}} \geq 1, \\ \frac{E_{\text{old}}}{E_{\text{new}}} T_A & \text{if } E_{\text{old}}/E_{\text{new}} < 1. \end{cases}$$

where $T_A = \dfrac{0.8}{\sqrt{1+i}}$; this particular form for T_A was chosen after an initial trial-and-error stage. This means that the probability of accepting changes that increase the error decreased with each annealing step. A typical run for our model ran this process for $10,000$ steps, after which the variable i was reset to zero and the process was started again, using as initial condition the final parameters of the previous run. This process was repeated 10 times, to ensure the process did not get trapped in suboptimal

minima. We then used the parameter configuration that minimized the energy function from among all visited configurations.

After obtaining a stable set of parameters for Eqs. (3), a few parameters in the model were close to 0. New fits were made forcing these parameters to be zero, and the values obtained for the energy function were as good or even lower than those obtained considering the parameters free. The improvement in the fit when excluding these parameters can be explained by the increase in the efficiency of sampling the parameter space when its dimension is reduced. Finding this parameters consistent with zero in our fit does not mean that the interactions they represent do not exist, but rather that they are not important in our experimental conditions, or that their effect is already captured in an effective way by other parameters of the model. After exclusion of the parameters deemed negligible by our fitting procedure, the model given by Eqs. (3) is simplified to Eqs. (1). This final model was then fitted to the data using the simulated annealing procedure. Independent runs of this process (with the same initial conditions described above) converge to the same (or a very similar) set of parameters, shown in table S1.

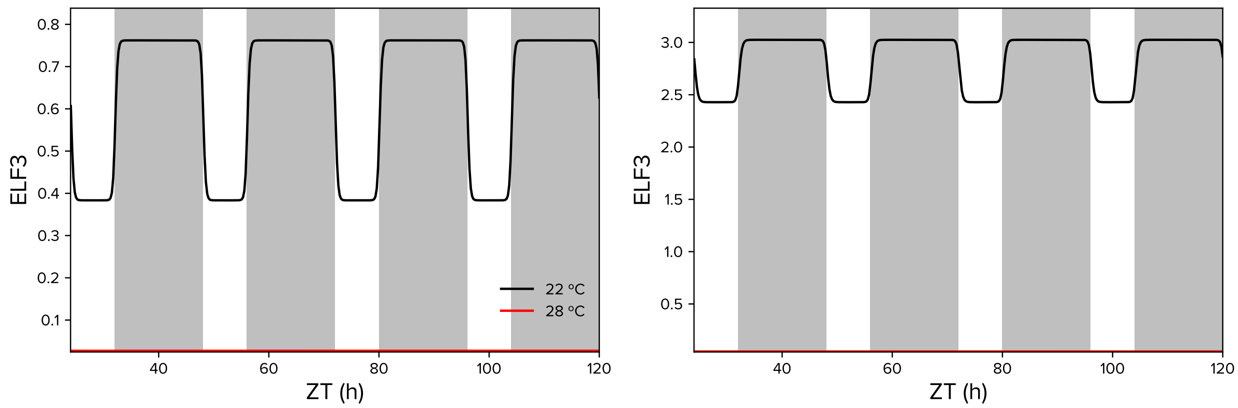

Figure S8: **ELF3 dynamics are not captured by the model if expression data is not included in the training dataset.** The two plots represent ELF3 dynamics from two parameter sets obtained in two simulated annealing runs where the expression data had no weight on the energy function ($w = 0$ in Eq. (4)). These two parameter sets can fit the growth data as well as the parameter set we have used in the main text (figures not shown). The result is that these ELF3 dynamics are not similar to the ones we observe experimentally.

The need for ELF3 expression

Ideally, we would like to use the least amount of data to fit our model, in order to increase its predictive capabilities. If a model trained only with growth data is able to predict with some precision expression timeseries, then it means that the model is somewhat accurate and we can use it for further predictions that we can test later. When we tried to do this, however, the model was not able to accurately reproduce ELF3 expression timeseries. We ran two simulated annealing runs with an energy function that did not give any weight to the expression data. In other words, we set $w = 0$ in Eq. (4). The resulting sets of parameters were able to fit the growth data as well as the final parameters used in the main text. However, they did not reproduce ELF3 expression accurately. In both cases, levels of ELF3 at 22°C were much higher than those at 28°C, which are almost null, fig. S8.

As a result, we decided to set $w = 1$ in Eq. (4), so as to force the model to reproduce the expression dynamics of ELF3.

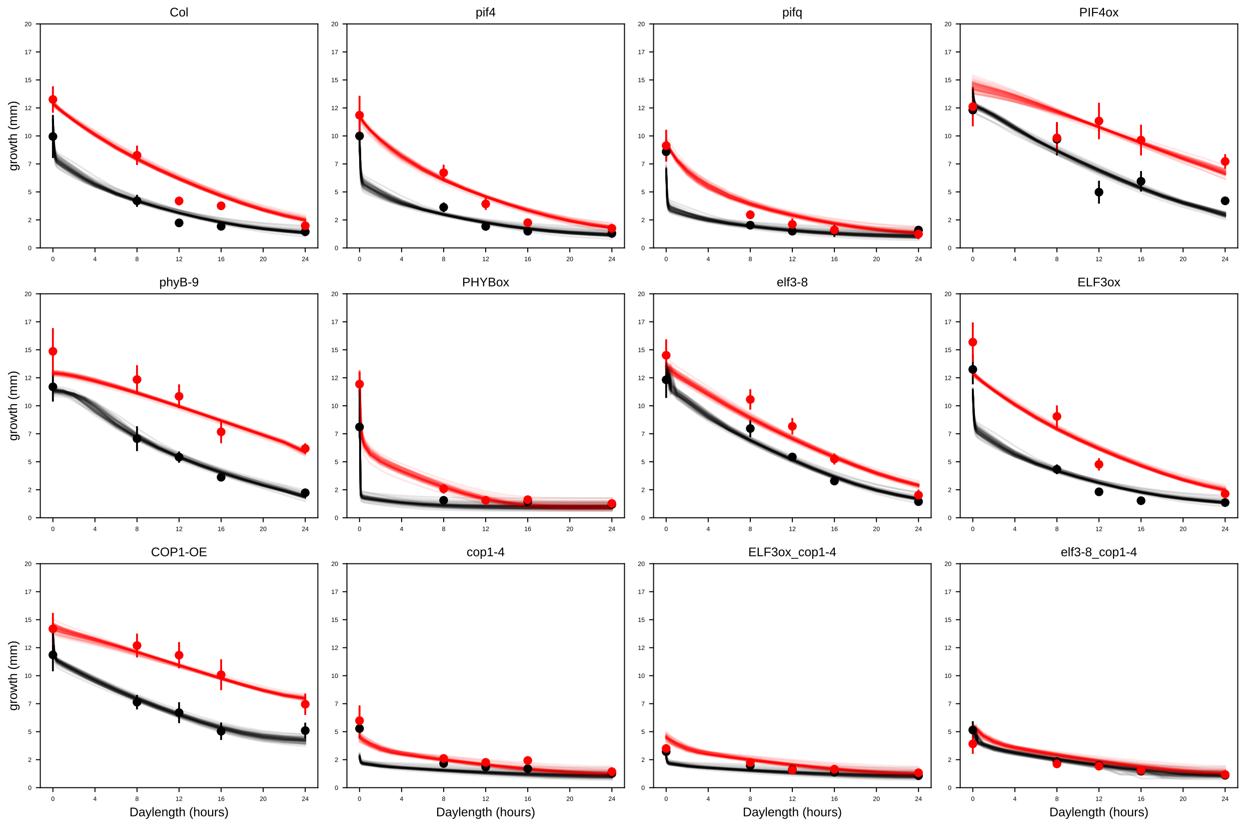

Figure S9: **Several parameter sets reproduce the growth data accurately.** The plot shows the growth predictions of 48 different parameter sets obtained from independent simulated annealing runs (solid lines). Most lines overlap and their discrepancies are small in comparison with the experimental variance of the dataset (filled circles are averages, lines represent one standard deviation).

Robustness of the fit

We ran several independent runs of the simulated annealing algorithm, where the initial values for the parameters were chosen at random from a normal distribution of mean 1 and standard deviation 0.1 (except those that were fixed at the beginning, as explained above). We obtained 48 parameter sets (including the one we used in the main text) that accurately reproduced our growth data, fig. S9. Parameter values, however, varied widely between fits fig. S10.

The explanation for this is that the optimal set of parameters lies on a high-dimensional manifold. This is especially clear in the case of the ELF3 parameters, which lie on a one-dimensional manifold in $\mathbb{R}^5$, fig. S11. In fact, the line given by the equation

$$p_{E1}(22) = 1.806 p_{E1}(28) - 0.759 p_{E2}(22) - 0.611 p_{E2}(28) - 3.213 d_E - 0.616$$

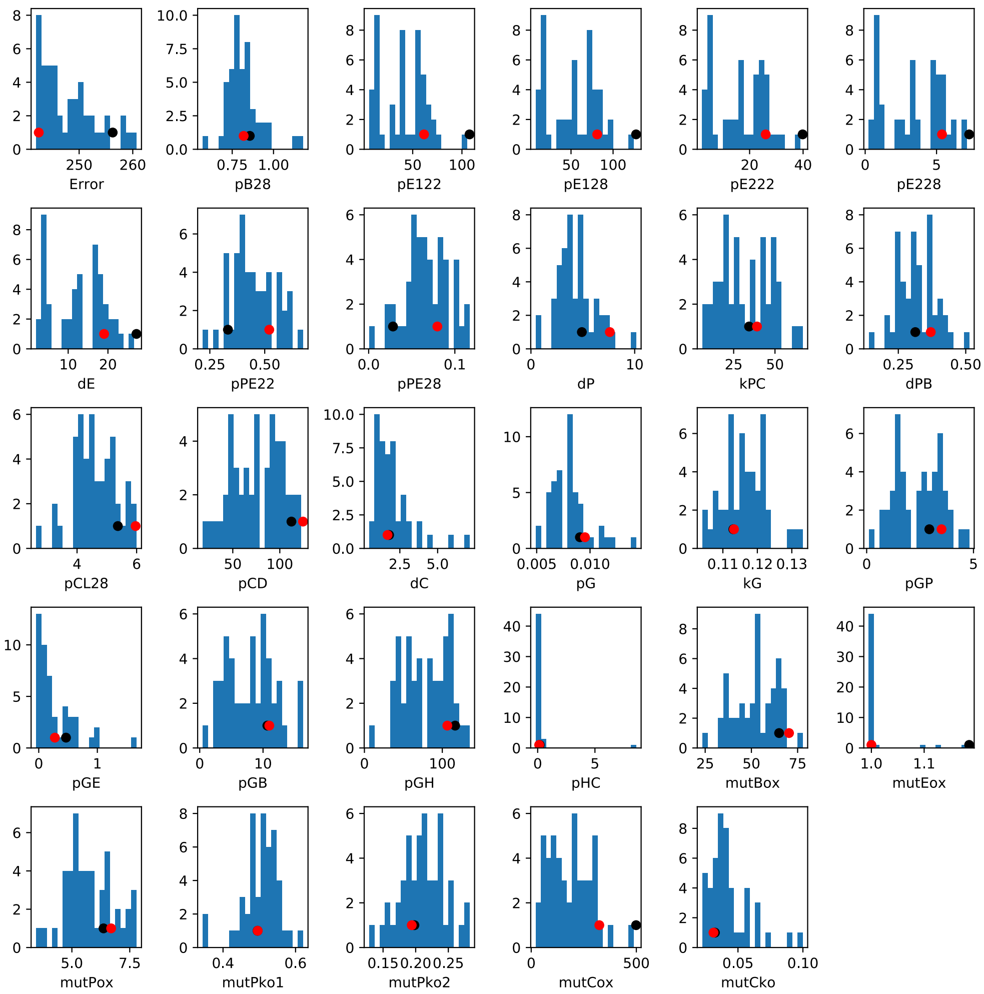

Figure S10: **Parameter values vary widely among parameter sets.** Histograms of all parameters in the model (including the energy function, first subplot)) made from 48 independent parameter sets that accurately fit our growth data. y axes represent histogram counts, while x axes represent parameter values. While some paremeters are more restricted in their values, most of them have a wide range of variation. Red dots mark the value of the parameter corresponding to the set that has a minimal energy function, while black dots represent the value of the parameter corresponding to the set used in the main text, table S1. Parameter labels: *mutBox* represents the multiplier associated with phyB's overexpressor line (and similarly with *mutCox, mutEox* and *mutPox*), *mutPko1* and *mutPko2* represent the multiplier associated with *pif4* and *pifq*, respectively, while *mutCko* represents the multiplier associated with *cop1-4*.

fits the five-dimensional data extremely well ($R^2 = 0.9997$). If we had experimental information to fix one of these ELF3 parameters, the others would be automatically determined. In the case of parameters

related to other variables, the relationships are harder to infer, due to their nonlinear nature and the high dimensionality of the problem.

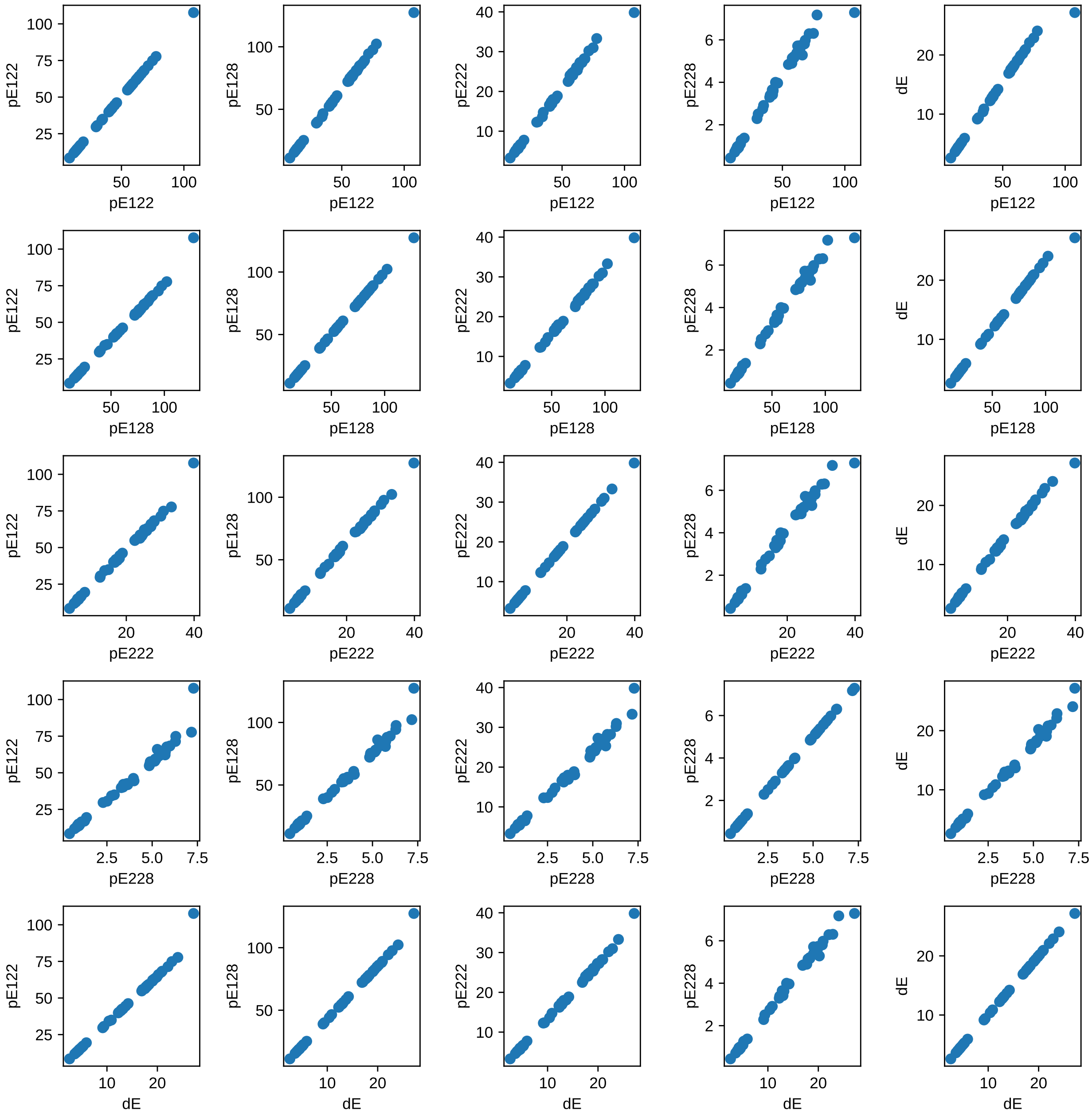

Figure S11: **ELF3 parameters lie on a five dimensional line in $\mathbb{R}^5$.** Parameters $pE1(22)$, $p_{E1}(28)$, $p_{E2}(22)$, $p_{E2}(28)$ and d_E vary widely among parameter sets that reproduce the growth data, fig. S10. However, their values are highly correlated: representing the values of one parameter in all sets against the values of another parameter, we see that the points lie on lines for all parameter combinations. This means that they are actually lying on a five dimensional line, which we can find numerically (see text).

It is not the case that, given a parameter set, we can change one of the parameter values within the observed range of variation in fig. S10 with complete freedom. This can be checked with a sensitivity analysis, where we study the change in the energy function due to a small perturbation in each of the parameters, fig. S12. More precisely, we estimate the partial derivative of the energy function $E(\theta_1,\ldots,\theta_n)$ in Eq. (4) with respect to each of the parameters θ_i using the central finite difference, that is

$$\frac{\partial E}{\partial \theta_i} \approx \frac{E(\ldots,\theta_i+h,\ldots)-E(\ldots,\theta_i-h,\ldots)}{2h}, \tag{5}$$

where we have used $h = 5\cdot 10^{-5} * \theta_i$ (i.e. a 0.005% change in θ_i), although the results are not dependent on the exact value of h. We then normalize this approximate derivative by the value of E at the original parameter set, in order to represent this sensitivity relative to the value of E.

While some parameters can be changed for some parameter sets, thus informing us that the fit could be improved, every parameter has a non-zero effect on E for at least one parameter set. In some cases this change is quite large, even greater than 100%. This informs us that all remaining parameters in the model are necessary and cannot be removed without incurring a cost in our ability to reproduce the growth data.

Different model topologies

In minimizing our energy function, we found that some parameters in the original model Eqs. (3) were close to zero, and so we removed them from the model for computational purposes. But is this final topology of interactions between molecular species the only one that can fit the data? The number of combinations of possible topologies is astronomically high, but we can try to answer this question by performing some tests. We can set to zero one of the nonzero parameters in the final model Eqs. (1), and substitute it for one of the parameters that was discarded from the original model Eqs. (3). We have done this three times:

1. We set k_r to zero in the dB/dt equation of Eqs. (1), and include the terms d_{BC} and d_{BP} discarded from Eqs. (3). The modified equation for phyB is then

   $$\frac{dB(t)}{dt} = p_B(T)L(t)(mut_B - B(t)) - d_{BC}B(t)C(t) - d_{BP}B(t)P(t).$$

   That is, we neglect the thermal reversion rate in phyB and instead model the decay of phyB in dependence with PIF4 and COP1.

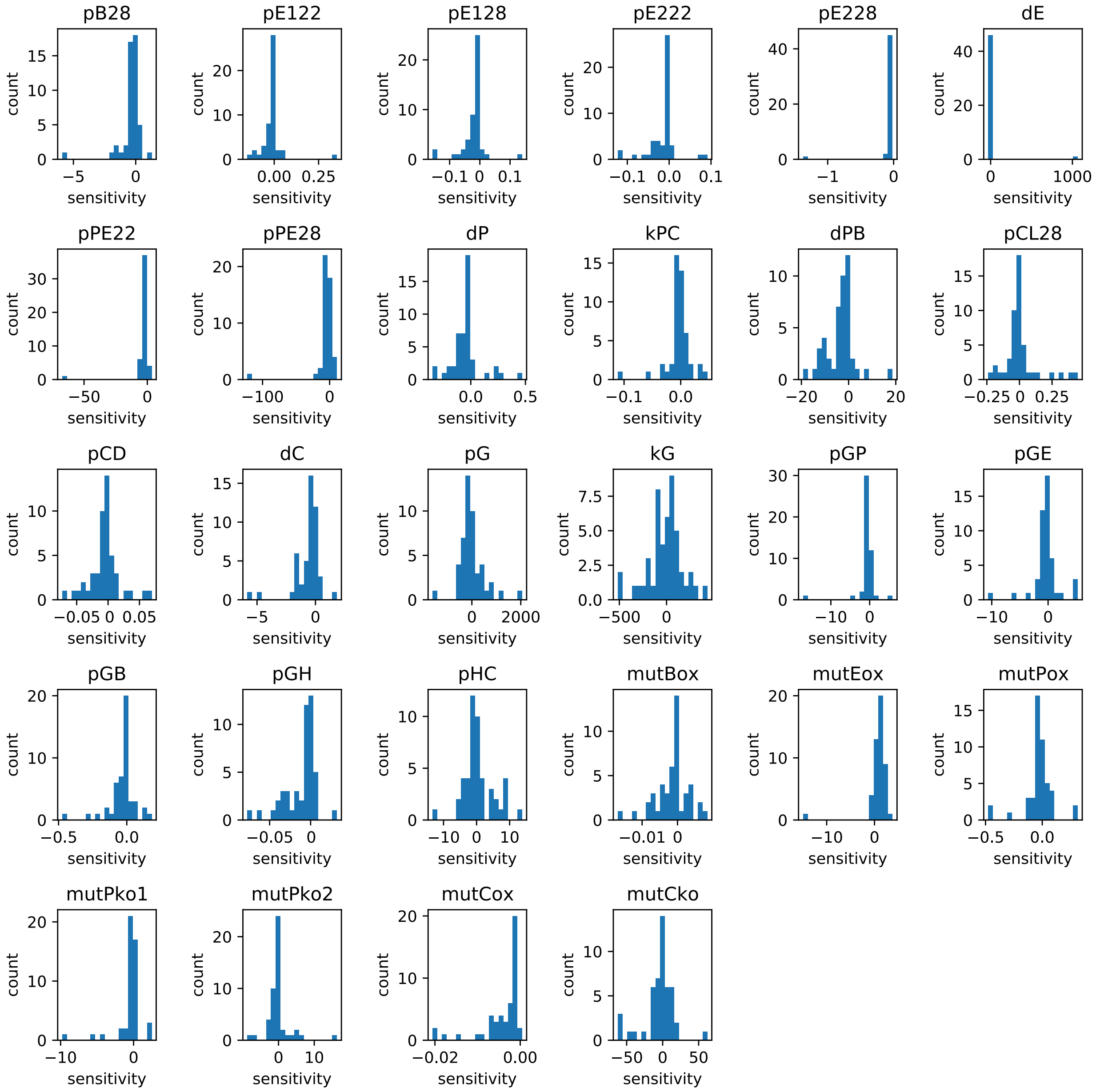

Figure S12: **Sensitivity of the energy function to small changes in the parameters.** Histograms showing sensitivity in the energy function E due to small changes in each of the parameters, Eq. (5). Each subplot represents small changes in one parameter, listed in the subtitle. Histograms made from 48 independent parameter sets that accurately fit our growth data. y axes represent histogram counts, while x axes represent percent sensitivity. Small changes in some parameters can cause very large changes in the energy function.

2. We set d_E to zero in the dE/dt equation of Eqs. (1), and include the term d_{EC} that was discarded

a) kr=0, dBC and dBP not zero

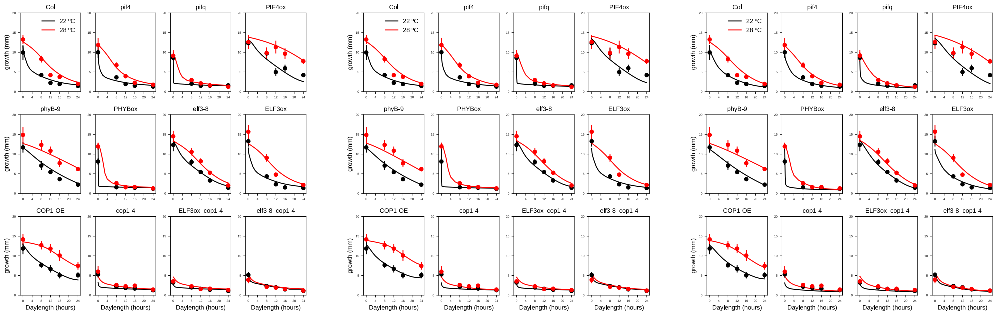

b) dE=0, dEC not zero

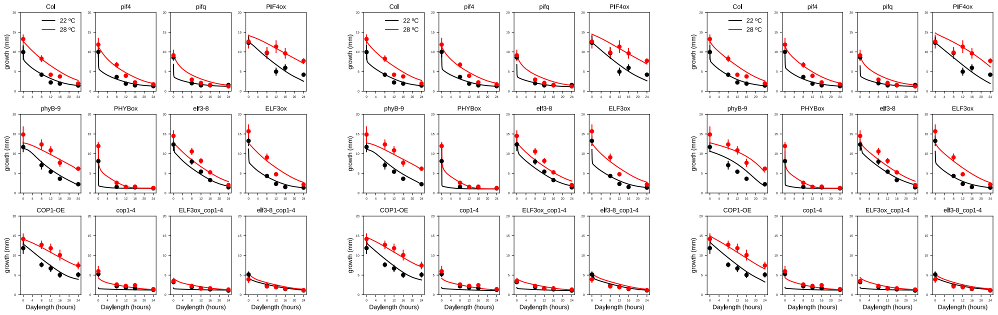

c) dC=0, dCB not zero

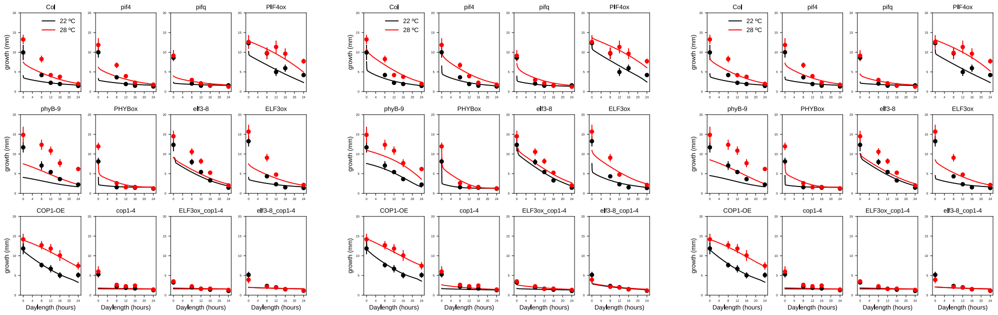

Figure S13: **Alternative network topologies can reproduce the growth data.** We performed nine simulated annealing runs with three alternative models (see text for an extended explanation), obtaining nine parameter sets that were able (in some cases) to reproduce the growth data as well as the final model Eqs. (1). **(Upper row)** Setting $k_r = 0$ and including in the model the terms d_{BC} and d_{BP} results in fits that reproduce the data very well. **(Middle row)** Similarly, setting $d_E = 0$ and including in the model the term d_{EC} results in a very good fit of the data, visually indistinguishable from the fits obtained in fig. S9. **(Lower row)** Setting $d_C = 0$ and including in the model the term d_{CB} from Eqs. (3) results in fits that do not reproduce the data accurately (notice in particular the bad fits of *Col, pif4, phyB-9, elf3-8 and ELF3ox*.

from Eqs. (3). The modified equation for ELF3 is then

$$\frac{dE(t)}{dt} = p_E(t,T,D,mut_E) - d_{EC}E(t)C(t).$$

That is, we neglect the decay of ELF3 due to any reason except its interaction with COP1.

3. We set d_C to zero in the dC/dt equation in Eqs. (3), and include the term d_{CB} that was discarded from Eqs. (3). The modified equation for COP1 is then

$$\frac{dC(t)}{dt} = mut_C\left[p_{CL}(T)L(t) + p_{CD}(1-L(t))\right] - d_{CB}C(t)B(t).$$

That is, we neglect the decay of COP1 due to any reason except its interaction with phyB.

We performed three simulated annealing runs with each of the modified models, thus obtained nine parameter sets. This is not an exhaustive computational experiment by any means, but it serves to test the hypothesis that these alternative models could fit the data as well as our final model Eqs. (1). The results are very instructive:

1. Setting $k_r = 0$ and allowing the model to change both d_{BC} and d_{BP} results in fits that are visually indistinguishable from Eqs. (1) at predicting the growth data (predictions shown in fig. S13, upper row). Whereas the minimum value of the energy function E is 242 in fig. S9, here we obtain $E < 237$ for all three parameter sets. However, we cannot conclude from this that the dark reversion of phyB does not occur. Indeed, this molecular process has been extensively documented and is essential in understanding phyB's dynamics. These results simply show that, mathematically, a model including phyB's degradation by both PIFs and COP1 is enough to reproduce our growth data. The reason for this is that all three terms are correlated: the role of phyB's dark reversion is more relevant during the night. The same happens with PIF and COP1-mediated degradation: as the activity of these proteins is higher during the night, so too is their inhibitory effect on phyB. The three terms therefore send a coherent signal downstream: phyB levels go down during the night. This coherence makes sense from the biological point of view, as systems benefit from being robust and typically have many redundant components (56). However, from the mathematical point of view, this means that the model has some flexibility in the sense of accepting different terms that act in the same direction.

2. Setting $d_E = 0$ and allowing the model to change d_{EC} results in fits that are slightly worse than the original model at capturing the growth data (predictions shown in fig. S13, middle row). However, visually there is no big difference with the fits we found for the simple model (compare

with fig. S9). Since the energy function is slightly higher ($E = 256$ and $E = 259$ for the two left subplots in the middle row of fig. S13), it makes sense that the simulated annealing algorithm "chooses" to discard d_{EC} and focuses on a fit using d_E alone. Note that values of the energy function for the simple model Eqs. (1) are on average lower than 250 (fig. S10, first subplot). However, from a biological standpoint, the difference is not high enough for us to claim that COP1-mediated degradation of ELF3 is not relevant in our system. We would need additional studies to separate the effect of COP1 and other elements that can contribute to the decay of ELF3 protein levels. As in the previous example, the model shows some flexibility when incorporating terms that act in the same direction.

3. Finally, setting $d_C = 0$ and allowing the model to change d_{CB} results in fits that do not capture the growth data accurately enough, with values of the energy function higher than 280 in all three cases (fig. S13, lower row). Notice for instance the bad fits of *Col, pif4, phyB-9* or *elf3-8* mutants. This suggests that the removal of COP1 from the nucleus is dependent not only on phyB but also on other photoreceptors, in line with our experimental data, fig. S5.

In summary, a closer inspection of alternative topologies allows us to conclude that COP1 dynamics are not mediated by phyB alone, and needs additional elements. However, we cannot conclude that COP1 and PIF-mediated inhibition of phyB is biologically irrelevant, or that COP1-mediated degradation of ELF3 does not have a role in the regulation of hypocotyl growth. In its current version, the model accepts some flexibility regarding these terms, as they all act in a correlated manner. In further works we will develop and impove the model in order to discern these contributions.

Heatmaps

As a final robustness test, we recreated the thermoelongation heatmaps in Fig. 6 (main text) for several parameter sets, in order to check whether model predictions were consistent. Results are shown in figs. S14, S15, and S16. The heatmaps change quantitatively (as expected), but not qualitatively. The "blue" and "red" regions remain invariant, and only some minor quantitative features change. The consistency of predictions among parameter sets proves that our results are not dependent on specific parameter values, thus supporting our main conclusions.

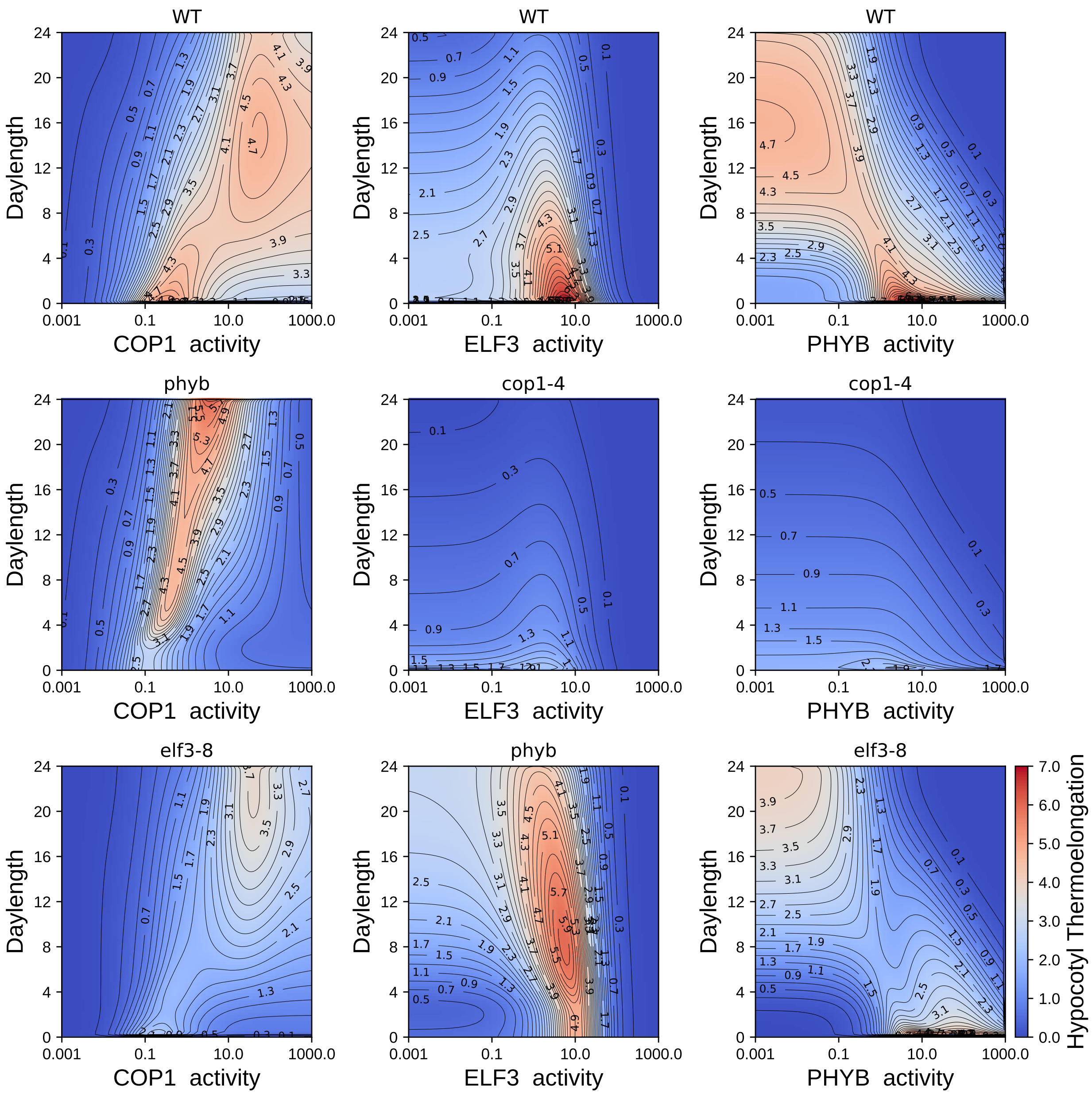

Figure S14: **Heatmap plots representing hypocotyl thermoelongation for several genotypes under different experimental conditions (I).** As in Fig. 6 (main text), but re-created using a different parameter set.

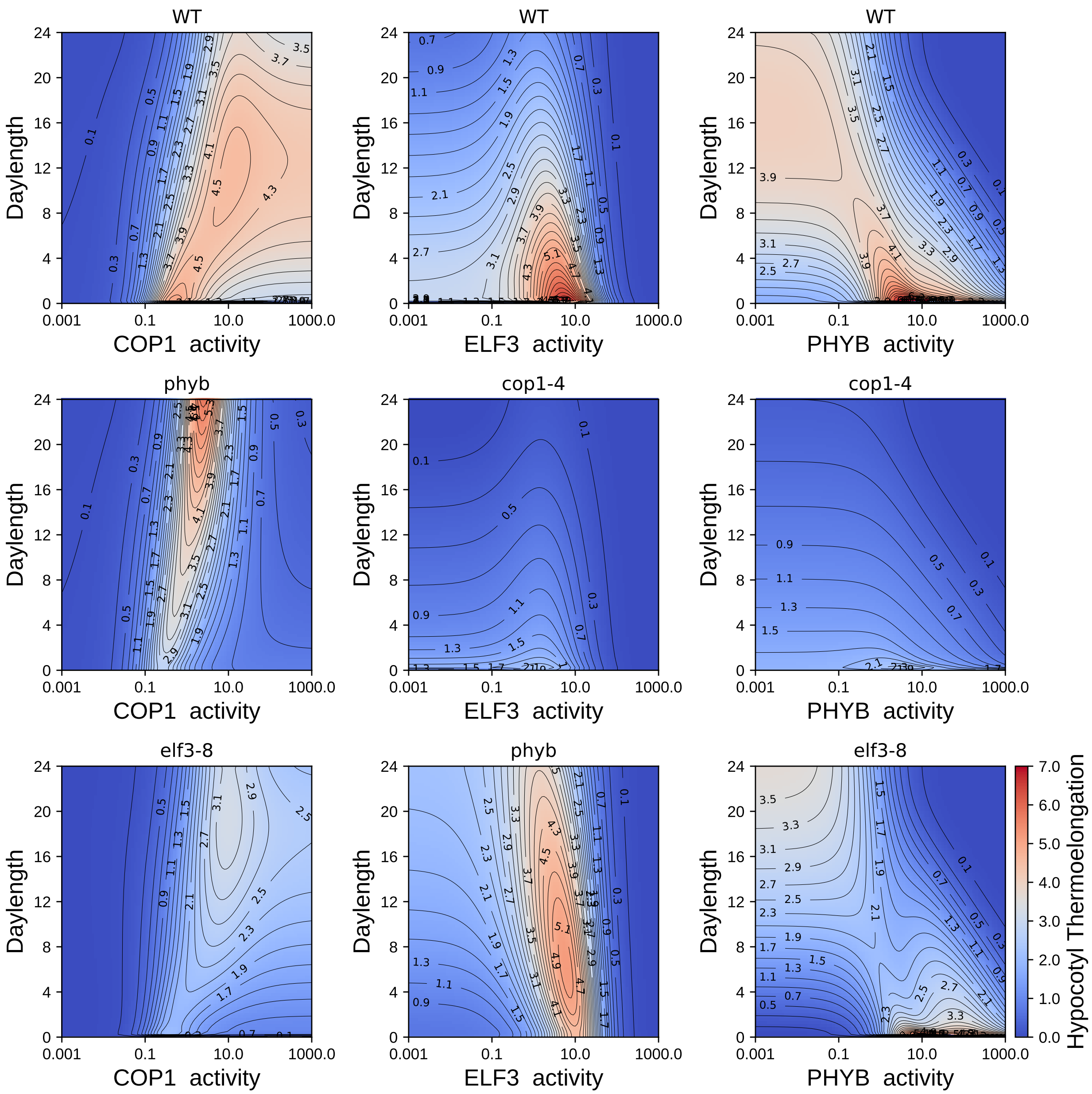

Figure S15: **Heatmap plots representing hypocotyl thermoelongation for several genotypes under different experimental conditions (II).** As in Fig. 6 (main text), but re-created using a different parameter set.

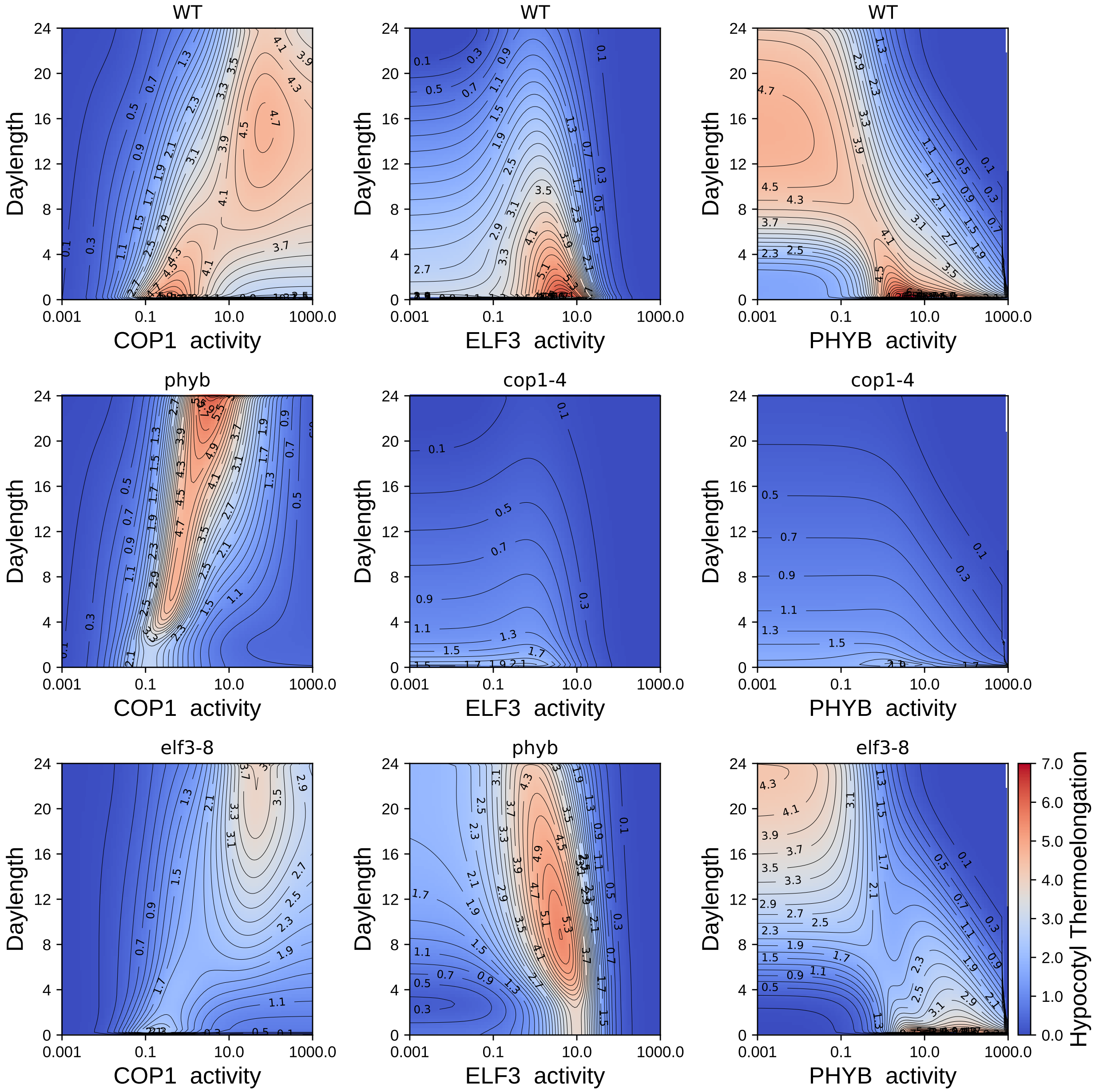

Figure S16: **Heatmap plots representing hypocotyl thermoelongation for several genotypes under different experimental conditions (III).** As in Fig. 6 (main text), but re-created using a different parameter set.

Supplementary Tables

Table S1 shows the parameters used for the simulation results shown in the main text. Tables S2 to S6 show the number of seedlings used to quantify each experimental condition.

Table S1: **Parameters used in Eq.** (1). **The unit used for time is hours, concentrations are taken as non-dimensional.**

$p_B(22)$	10.0	$p_B(28)$	0.860
$k_r(22)$	0.232	$k_r(28)$	0.411
$p_{E1}(22)$	108	$p_{E1}(28)$	127
$p_{E2}(22)$	39.8	$p_{E2}(28)$	7.29
d_E	27.2		
$p_{CL}(22)$	1.00	$p_{CL}(28)$	5.37
p_{CD}	112	d_C	1.79
p_P	1	d_{PB}	0.313
$p_{PE}(22)$	0.332	$p_{PE}(28)$	0.028
d_P	4.91	k_{PC}	34.3
p_G	0.009	k_G	0.113
p_{GP}	2.93	p_{GE}	0.465
p_{GB}	10.7	p_{GH}	116
p_{HC}	0.180		
$mut_B(phyB-9)$	0	$mut_B(PHYBox)$	65.0
$mut_B(elf3-8)$	0	$mut_E(ELF3ox)$	1.18
$mut_C(cop1-4)$	0.032	$mut_C(COP1-OE)$	499
$mut_P(cop1-6)$	0.056		
$mut_P(pif4)$	0.495	$mut_P(PIF4ox)$	6.37
$mut_P(pifq)$	0.198		

Table S2: **Datapoints used to generate Fig. 1 and fig. S4, for every experimental treatment measured. Seedlings of ELF3ox at 28ºC and 16 hours of light and PHYBox at 22ºC and 12 hours of light failed to germinate. Experiments of these conditions on different sets produced results as expected with the behavior in Fig. 1, but were not included for consistency of the dataset.**

Genotype	Daylength	Temperature	Datapoints
Col	0	22	20
Col	0	28	20
Col	8	22	19
Col	8	28	18
Col	12	22	15
Col	12	28	19
Col	16	22	30
Col	16	28	25
Col	24	22	13
Col	24	28	11
cop1-4	0	22	31
cop1-4	0	28	30
cop1-4	8	22	29
cop1-4	8	28	33
cop1-4	12	22	21
cop1-4	12	28	20
cop1-4	16	22	25
cop1-4	16	28	29
cop1-4	24	22	26
cop1-4	24	28	26
cop1-6	0	22	51
cop1-6	0	28	59
cop1-6	8	22	14
cop1-6	8	28	13
cop1-6	12	22	36
cop1-6	12	28	37
cop1-6	16	22	34
cop1-6	16	28	38
cop1-6	24	22	37
cop1-6	24	28	41
COP1-OE	0	22	74
COP1-OE	0	28	71
COP1-OE	8	22	14
COP1-OE	8	28	15
COP1-OE	12	22	43
COP1-OE	12	28	52
COP1-OE	16	22	74
COP1-OE	16	28	74
COP1-OE	24	22	56
COP1-OE	24	28	60
elf3-8	0	22	35
elf3-8	0	28	31
elf3-8	8	22	11
elf3-8	8	28	31
elf3-8	12	22	22
elf3-8	12	28	25
elf3-8	16	22	9
elf3-8	16	28	16
elf3-8	24	22	28
elf3-8	24	28	25
elf3-8 cop1-4	0	22	34
elf3-8 cop1-4	0	28	35
elf3-8 cop1-4	8	22	32
elf3-8 cop1-4	8	28	35
elf3-8 cop1-4	12	22	23
elf3-8 cop1-4	12	28	27
elf3-8 cop1-4	16	22	10
elf3-8 cop1-4	16	28	11
elf3-8 cop1-4	24	22	25
elf3-8 cop1-4	24	28	19
elf3-8 phyB-9	0	22	32
elf3-8 phyB-9	0	28	27
elf3-8 phyB-9	8	22	35
elf3-8 phyB-9	8	28	37
elf3-8 phyB-9	12	22	27
elf3-8 phyB-9	12	28	36
elf3-8 phyB-9	16	22	31
elf3-8 phyB-9	16	28	34
elf3-8 phyB-9	24	22	36
elf3-8 phyB-9	24	28	34
ELF3ox	0	22	34
ELF3ox	0	28	35
ELF3ox	8	22	33
ELF3ox	8	28	35
ELF3ox	12	22	17
ELF3ox	12	28	22
ELF3ox	16	22	22
ELF3ox	16	28	0
ELF3ox	24	22	27
ELF3ox	24	28	31
ELF3ox cop1-4	0	22	31
ELF3ox cop1-4	0	28	31
ELF3ox cop1-4	8	22	35
ELF3ox cop1-4	8	28	29
ELF3ox cop1-4	12	22	36
ELF3ox cop1-4	12	28	25
ELF3ox cop1-4	16	22	19
ELF3ox cop1-4	16	28	24
ELF3ox cop1-4	24	22	25
ELF3ox cop1-4	24	28	20
hy5	0	22	24
hy5	0	28	15
hy5	8	22	21
hy5	8	28	19
hy5	12	22	14
hy5	12	28	11
hy5	16	22	13
hy5	16	28	16
hy5	24	22	14
hy5	24	28	14
phyB-9	0	22	15
phyB-9	0	28	17
phyB-9	8	22	23
phyB-9	8	28	22
phyB-9	12	22	17
phyB-9	12	28	16
phyB-9	16	22	4
phyB-9	16	28	11
phyB-9	24	22	19
phyB-9	24	28	17
phyB-9 cop1-4	0	22	28
phyB-9 cop1-4	0	28	24
phyB-9 cop1-4	8	22	38
phyB-9 cop1-4	8	28	35
phyB-9 cop1-4	12	22	not measured
phyB-9 cop1-4	12	28	not measured
phyB-9 cop1-4	16	22	34
phyB-9 cop1-4	16	28	32
phyB-9 cop1-4	24	22	24
phyB-9 cop1-4	24	28	25
PHYBox	0	22	16
PHYBox	0	28	15
PHYBox	8	22	12
PHYBox	8	28	11
PHYBox	12	22	0
PHYBox	12	28	3
PHYBox	16	22	2
PHYBox	16	28	6
PHYBox	24	22	2
PHYBox	24	28	4
pif4	0	22	21
pif4	0	28	27
pif4	8	22	27
pif4	8	28	29
pif4	12	22	29
pif4	12	28	26
pif4	16	22	15
pif4	16	28	15
pif4	24	22	18
pif4	24	28	19
PIF4ox	0	22	1
PIF4ox	0	28	5
PIF4ox	8	22	8
PIF4ox	8	28	10
PIF4ox	12	22	12
PIF4ox	12	28	8
PIF4ox	16	22	14
PIF4ox	16	28	20
PIF4ox	24	22	4
PIF4ox	24	28	15
pifq	0	22	6
pifq	0	28	7
pifq	8	22	10
pifq	8	28	16
pifq	12	22	5
pifq	12	28	8
pifq	16	22	2
pifq	16	28	3
pifq	24	22	2
pifq	24	28	3

Table S3: **Datapoints used to generate Fig. 3, for every experimental treatment measured.**

Genotype	Daylength	Light	Temperature	Datapoints
WT	4	White	22	33
WT	4	White	28	31
phyB-9	4	White	22	28
phyB-9	4	White	28	24
PHYBox	4	White	22	58
PHYBox	4	White	28	56
COP1-OE	4	White	22	29
COP1-OE	4	White	28	26
cop1-4	4	White	22	19
cop1-4	4	White	28	19
elf3-8	4	White	22	31
elf3-8	4	White	28	37

Table S4: **Datapoints used to generate Fig. 5C, for every experimental treatment measured.**

Genotype	Daylength	Light	Temperature	Datapoints
WT	24	White	22	23
WT	24	White	28	21
YFP-COP1	24	White	22	15
YFP-COP1	24	White	28	14
RFP-COP1	24	White	22	24
RFP-COP1	24	White	28	24
COP1-OE	24	White	22	24
COP1-OE	24	White	28	22
cop1-4	24	White	22	19
cop1-4	24	White	28	19
WT	0	White	22	14
WT	0	White	28	21
YFP-COP1	0	White	22	18
YFP-COP1	0	White	28	18
RFP-COP1	0	White	22	24
RFP-COP1	0	White	28	22
COP1-OE	0	White	22	23
COP1-OE	0	White	28	22
cop1-4	0	White	22	18
cop1-4	0	White	28	19

Table S5: **Datapoints used to generate Fig. 5G and fig. S5B, for every experimental treatment measured.**

Genotype	Daylength	Light	Temperature	Datapoints
PHYBox COP1OE	0		22	63
PHYBox COP1OE	0		28	57
PHYBox COP1OE	24	White	22	17
PHYBox COP1OE	24	White	28	34
PHYBox COP1OE	24	RED	22	26
PHYBox COP1OE	24	RED	28	20
PHYBox COP1OE	24	BLUE	22	30
PHYBox COP1OE	24	BLUE	28	31
phyB COP1 OE	0		22	111
phyB COP1 OE	0		28	87
phyB COP1 OE	24	White	22	112
phyB COP1 OE	24	White	28	91
phyB COP1 OE	24	RED	22	37
phyB COP1 OE	24	RED	28	28
phyB COP1 OE	24	BLUE	22	22
phyB COP1 OE	24	BLUE	28	20
PHYBox COP1OE	4	White	22	33
PHYBox COP1OE	4	White	28	30
PHYBox COP1OE	8	White	22	21
PHYBox COP1OE	8	White	28	16
PHYBox COP1OE	16	White	22	17
PHYBox COP1OE	16	White	28	18
phyB COP1 OE	4	White	22	52
phyB COP1 OE	4	White	28	44
phyB COP1 OE	8	White	22	13
phyB COP1 OE	8	White	28	26
phyB COP1 OE	16	White	22	39
phyB COP1 OE	16	White	28	42

Table S6: **Datapoints used to generate fig. S5A, for every experimental treatment measured.**

Genotype	Daylength	Light	Temperature	Datapoints
WT	24	BLUE	22	39
WT	24	BLUE	28	46
YFP-COP1	24	BLUE	22	31
YFP-COP1	24	BLUE	28	27
RFP-COP1	24	BLUE	22	46
RFP-COP1	24	BLUE	28	48
COP1-OE	24	BLUE	22	43
COP1-OE	24	BLUE	28	46
WT	24	RED	22	44
WT	24	RED	28	49
YFP-COP1	24	RED	22	31
YFP-COP1	24	RED	28	31
RFP-COP1	24	RED	22	42
RFP-COP1	24	RED	28	35
COP1-OE	24	RED	22	57
COP1-OE	24	RED	28	46

REFERENCES AND NOTES

1. E. S. Burgie, R. D. Vierstra, Phytochromes: An atomic perspective on photoactivation and signaling. *Plant Cell* **26**, 4568–4583 (2014).

2. J. J. Casal, Photoreceptor signaling networks in plant responses to shade. *Annu. Rev. Plant Biol.* **64**, 403–427 (2013).

3. C. M. M. Gommers, E. J. W. Visser, K. R. S. Onge, L. A. C. J. Voesenek, R. Pierik, Shade tolerance: When growing tall is not an option. *Trends Plant Sci.* **18**, 65–71 (2013).

4. J.-H. Jung, M. Domijan, C. Klose, S. Biswas, D. Ezer, M. Gao, A. K. Khattak, M. S. Box, V. Charoensawan, S. Cortijo, M. Kumar, A. Grant, J. C. W. Locke, E. Schäfer, K. E. Jaeger, P. A. Wigge, Phytochromes function as thermosensors in Arabidopsis. *Science* **354**, 886–889 (2016).

5. M. Legris, C. Klose, E. S. Burgie, C. C. R. Rojas, M. Neme, A. Hiltbrunner, P. A. Wigge, E. Schäfer, R. D. Vierstra, J. J. Casal, Phytochrome B integrates light and temperature signals in Arabidopsis. *Science* **354**, 897–900 (2016).

6. J.-H. Jung, A. D. Barbosa, S. Hutin, J. R. Kumita, M. Gao, D. Derwort, C. S. Silva, X. Lai, E. Pierre, F. Geng, S.-B. Kim, S. Baek, C. Zubieta, K. E. Jaeger, P. A. Wigge, A prion-like domain in ELF3 functions as a thermosensor in Arabidopsis. *Nature* **585**, 256–260 (2020).

7. D. Ezer, J.-H. Jung, H. Lan, S. Biswas, L. Gregoire, M. S. Box, V. Charoensawan, S. Cortijo, X. Lai, D. Stöckle, C. Zubieta, K. E. Jaeger, P. A. Wigge, The evening complex coordinates environmental and endogenous signals in Arabidopsis. *Nat. Plants* **3**, 17087 (2017).

8. C. S. Silva, A. Nayak, X. Lai, S. Hutin, V. Hugouvieux, J.-H. Jung, I. López-Vidriero, J. M. Franco-Zorrilla, K. C. S. Panigrahi, M. H. Nanao, P. A. Wigge, C. Zubieta, Molecular mechanisms of evening complex activity inArabidopsis. *Proc. Natl. Acad. Sci. U.S.A.* **117**, 6901–6909 (2020).

9. M. S. Box, B. E. Huang, M. Domijan, K. E. Jaeger, A. K. Khattak, S. J. Yoo, E. L. Sedivy, D. M. Jones, T. J. Hearn, A. A. R. Webb, A. Grant, J. C. W. Locke, P. A. Wigge, ELF3 controls thermoresponsive growth in Arabidopsis. *Curr. Biol.* **25**, 194–199 (2015).

10. M. A. Koini, L. Alvey, T. Allen, C. A. Tilley, N. P. Harberd, G. C. Whitelam, K. A. Franklin, High temperature-mediated adaptations in plant architecture require the bHLH transcription factor PIF4. *Curr. Biol.* **19**, 408–413 (2009).

11. A.-S. Fiorucci, V. C. Galvão, Y. Ç. Ince, A. Boccaccini, A. Goyal, L. Allenbach Petrolati, M. Trevisan, C. Fankhauser, PHYTOCHROME INTERACTING FACTOR 7 is important for early responses to elevated temperature in Arabidopsis seedlings. *New Phytol.* **226**, 50–58 (2020).

12. V. N. Pham, X. Xu, E. Huq, Molecular bases for the constitutive photomorphogenic phenotypes in *Arabidopsis*. *Development* **145**, dev169870 (2018).

13. E. Park, Y. Kim, G. Choi, Phytochrome B requires PIF degradation and sequestration to induce light responses across a wide range of light conditions. *Plant Cell* **30**, 1277–1292 (2018).

14. A. Helfer, D. A. Nusinow, B. Y. Chow, A. R. Gehrke, M. L. Bulyk, S. A. Kay, LUX ARRHYTHMO encodes a nighttime repressor of circadian gene expression in the Arabidopsis core clock. *Curr. Biol.* **21**, 126–133 (2011).

15. D. A. Nusinow, A. Helfer, E. E. Hamilton, J. J. King, T. Imaizumi, T. F. Schultz, E. M. Farré, S. A. Kay, The ELF4-ELF3-LUX complex links the circadian clock to diurnal control of hypocotyl growth. *Nature* **475**, 398–402 (2011).

16. M. T. Osterlund, N. Wei, X. W. Deng, The roles of photoreceptor systems and the COP1-targeted destabilization of HY5 in light control of Arabidopsis seedling development. *Plant Physiol.* **124**, 1520–1524 (2000).

17. P. D. Duek, M. V. Elmer, V. R. van Oosten, C. Fankhauser, The degradation of HFR1, a putative bHLH class transcription factor involved in light signaling, is regulated by phosphorylation and requires COP1. *Curr. Biol.* **14**, 2296–2301 (2004).

18. J. Yang, R. Lin, U. Hoecker, B. Liu, L. Xu, H. Wang, Repression of light signaling by Arabidopsis SPA1 involves post-translational regulation of HFR1 protein accumulation. *Plant J.* **43**, 131–141 (2005).

19. X.-D. Lu, C.-M. Zhou, P.-B. Xu, Q. Luo, H.-L. Lian, H.-Q. Yang, Red-light-dependent interaction of phyB with SPA1 promotes COP1-SPA1 dissociation and photomorphogenic development in Arabidopsis. *Mol. Plant* **8**, 467–478 (2015).

20. C. Subramanian, B.-H. Kim, N. N. Lyssenko, X. Xu, C. H. Johnson, A. G. von Arnim, The Arabidopsis repressor of light signaling, COP1, is regulated by nuclear exclusion: Mutational analysis by bioluminescence resonance energy transfer. *Proc. Natl. Acad. Sci. U.S.A.* **101**, 6798–6802 (2004).

21. P. Hornitschek, S. Lorrain, V. Zoete, O. Michielin, C. Fankhauser, Inhibition of the shade avoidance response by formation of non-DNA binding bHLH heterodimers. *EMBO J.* **28**, 3893–3902 (2009).

22. H. Shi, S. Zhong, X. Mo, N. Liu, C. D. Nezames, X. W. Deng, HFR1 sequesters PIF1 to govern the transcriptional network underlying light-initiated seed germination in Arabidopsis. *Plant Cell* **25**, 3770–3784 (2013).

23. D. Chen, G. Xu, W. Tang, Y. Jing, Q. Ji, Z. Fei, R. Lin, Antagonistic basic helix-loop-helix/bZIP transcription factors form transcriptional modules that integrate light and reactive oxygen species signaling in Arabidopsis. *Plant Cell* **25**, 1657–1673 (2013).

24. G. Toledo-Ortiz, H. Johansson, K. P. Lee, J. Bou-Torrent, K. Stewart, G. Steel, M. Rodríguez-Concepción, K. J. Halliday, The HY5-PIF regulatory module coordinates light and temperature control of photosynthetic gene transcription. *PLOS Genet.* **10**, e1004416 (2014).

25. Y.-J. Park, H.-J. Lee, J.-H. Ha, J. Y. Kim, C.-M. Park, COP1 conveys warm temperature information to hypocotyl thermomorphogenesis. *New Phytol.* **215**, 269–280 (2017).

26. J.-W. Yu, V. Rubio, N.-Y. Lee, S. Bai, S.-Y. Lee, S.-S. Kim, L. Liu, Y. Zhang, M. L. Irigoyen, J. A. Sullivan, Y. Zhang, I. Lee, Q. Xie, N.-C. Paek, X. W. Deng, COP1 and ELF3 control circadian function and photoperiodic flowering by regulating GI stability. *Mol. Cell* **32**, 617–630 (2008).

27. I.-C. Jang, R. Henriques, H. S. Seo, A. Nagatani, N.-H. Chua, Arabidopsis PHYTOCHROME INTERACTING FACTOR proteins promote phytochrome B polyubiquitination by COP1 E3 ligase in the nucleus. *Plant Cell* **22**, 2370–2383 (2010).

28. C. Nieto, V. López-Salmerón, J.-M. Davière, S. Prat, ELF3-PIF4 interaction regulates plant growth independently of the Evening Complex. *Curr. Biol.* **25**, 187–193 (2015).

29. B. Al-Sady, W. Ni, S. Kircher, E. Schäfer, P. H. Quail, Photoactivated phytochrome induces rapid PIF3 phosphorylation prior to proteasome-mediated degradation. *Mol. Cell* **23**, 439–446 (2006).

30. W. Ni, S.-L. Xu, J. M. Tepperman, D. J. Stanley, D. A. Maltby, J. D. Gross, A. L. Burlingame, Z.-Y. Wang, P. H. Quail, A mutually assured destruction mechanism attenuates light signaling in Arabidopsis. *Science* **344**, 1160–1164 (2014).

31. C. Delker, L. Sonntag, G. V. James, P. Janitza, C. Ibañez, H. Ziermann, T. Peterson, K. Denk, S. Mull, J. Ziegler, S. J. Davis, K. Schneeberger, M. Quint, The DET1-COP1-HY5 pathway constitutes a multipurpose signaling module regulating plant photomorphogenesis and thermomorphogenesis. *Cell Rep.* **9**, 1983–1989 (2014).

32. J.-J. Ling, J. Li, D. Zhu, X. W. Deng, Noncanonical role of *Arabidopsis* COP1/SPA complex in repressing BIN2-mediated PIF3 phosphorylation and degradation in darkness. *Proc. Natl. Acad. Sci. U.S.A.* **114**, 3539–3544 (2017).

33. V. N. Pham, P. K. Kathare, E. Huq, Phytochromes and phytochrome interacting factors. *Plant Physiol.* **176**, 1025–1038 (2018).

34. C. D. Crocco, A. Locascio, C. M. Escudero, D. Alabadí, M. A. Blázquez, J. F. Botto, The transcriptional regulator BBX24 impairs DELLA activity to promote shade avoidance in Arabidopsis thaliana. *Nat. Commun.* **6**, 6202 (2015).

35. H. Huang, S. Alvarez, R. Bindbeutel, Z. Shen, M. J. Naldrett, B. S. Evans, S. P. Briggs, L. M. Hicks, S. A. Kay, D. A. Nusinow, Identification of evening complex associated proteins in arabidopsis by affinity purification and mass spectrometry. *Mol. Cell. Proteomics* **15**, 201–217 (2016).

36. M. F. Covington, S. Panda, X. L. Liu, C. A. Strayer, D. R. Wagner, S. A. Kay, ELF3 modulates resetting of the circadian clock in Arabidopsis. *Plant Cell* **13**, 1305–1316 (2001).

37. R. Podolec, R. Ulm, Photoreceptor-mediated regulation of the COP1/SPA E3 ubiquitin ligase. *Curr. Opin. Plant Biol.* **45**, 18–25 (2018).

38. J. De Caluwé, Q. Xiao, C. Hermans, N. Verbruggen, J.-C. Leloup, D. Gonze, A compact model for the complex plant circadian clock. *Front. Plant Sci.* **7**, 74 (2016).

39. H. Johansson, H. J. Jones, J. Foreman, J. R. Hemsted, K. Stewart, R. Grima, K. J. Halliday, *Arabidopsis* cell expansion is controlled by a photothermal switch. *Nat. Commun.* **5**, 4848 (2014).

40. U. Hoecker, The activities of the E3 ubiquitin ligase COP1/SPA, a key repressor in light signaling. *Curr. Opin. Plant Biol.* **37**, 63–69 (2017).

41. T. Mizuno, Y. Nomoto, H. Oka, M. Kitayama, A. Takeuchi, M. Tsubouchi, T. Yamashino, Ambient temperature signal feeds into the circadian clock transcriptional circuitry through the EC night-time repressor in Arabidopsis thaliana. *Plant Cell Physiol.* **55**, 958–976 (2014).

42. T. Yamashino, Y. Nomoto, S. Lorrain, M. Miyachi, S. Ito, N. Nakamichi, C. Fankhauser, T. Mizuno, Verification at the protein level of the PIF4-mediated external coincidence model for the temperature-adaptive photoperiodic control of plant growth in *Arabidopsis thaliana*. *Plant Signal. Behav.* **8**, e23390 (2013).

43. S. Bernardo-García, M. de Lucas, C. Martínez, A. Espinosa-Ruiz, J.-M. Davière, S. Prat, BR-dependent phosphorylation modulates PIF4 transcriptional activity and shapes diurnal hypocotyl growth. *Genes Dev.* **28**, 1681–1694 (2014).

44. D. D. Seaton, R. W. Smith, Y. H. Song, D. R. MacGregor, K. Stewart, G. Steel, J. Foreman, S. Penfield, T. Imaizumi, A. J. Millar, K. J. Halliday, Linked circadian outputs control elongation growth and flowering in response to photoperiod and temperature. *Mol. Syst. Biol.* **11**, 776 (2015).

45. M. de Lucas, J.-M. Davière, M. Rodríguez-Falcón, M. Pontin, J. M. Iglesias-Pedraz, S. Lorrain, C. Fankhauser, M. A. Blázquez, E. Titarenko, S. Prat, A molecular framework for light and gibberellin control of cell elongation. *Nature* **451**, 480–484 (2008).

46. J. Hahm, K. Kim, Y. Qiu, M. Chen, Increasing ambient temperature progressively disassembles Arabidopsis phytochrome B from individual photobodies with distinct thermostabilities. *Nat. Commun.* **11**, 1660 (2020).

47. E. K. Van Buskirk, A. K. Reddy, A. Nagatani, M. Chen, Photobody localization of phytochrome B is tightly correlated with prolonged and light-dependent inhibition of hypocotyl elongation in the dark. *Plant Physiol.* **165**, 595–607 (2014).

48. E. K. Van Buskirk, P. V. Decker, M. Chen, Photobodies in light signaling. *Plant Physiol.* **158**, 52–60 (2012).

49. A. Oravecz, A. Baumann, Z. Máté, A. Brzezinska, J. Molinier, E. J. Oakeley, E. Adám, E. Schäfer, F. Nagy, R. Ulm, CONSTITUTIVELY PHOTOMORPHOGENIC1 is required for the UV-B response in *Arabidopsis*. *Plant Cell* **18**, 1975–1990 (2006).

50. X.-L. Lin, D. Niu, Z.-L. Hu, D. H. Kim, Y. H. Jin, B. Cai, P. Liu, K. Miura, D.-J. Yun, W.-Y. Kim, R. Lin, J. B. Jin, An Arabidopsis SUMO E3 ligase, SIZ1, negatively regulates photomorphogenesis by promoting COP1 activity. *PLOS Genet.* **12**, e1006016 (2016).

51. D. J. Sheerin, C. Menon, S. zur Oven-Krockhaus, B. Enderle, L. Zhu, P. Johnen, F. Schleifenbaum, Y.-D. Stierhof, E. Huq, A. Hiltbrunner, Light-activated phytochrome A and B interact with members of the SPA family to promote photomorphogenesis in Arabidopsis by reorganizing the COP1/SPA complex. *Plant Cell* **27**, 189–201 (2015).

52. B. Liu, Z. Zuo, H. Liu, X. Liu, C. Lin, Arabidopsis cryptochrome 1 interacts with SPA1 to suppress COP1 activity in response to blue light. *Genes Dev.* **25**, 1029–1034 (2011).

53. Z. Zuo, H. Liu, B. Liu, X. Liu, C. Lin, Blue light-dependent interaction of CRY2 with SPA1 regulates COP1 activity and floral initiation in Arabidopsis. *Curr. Biol.* **21**, 841–847 (2011).

54. J. Ponnu, Molecular mechanisms suppressing COP1/SPA E3 ubiquitin ligase activity in blue light. *Physiol. Plant.* **169**, 418–429 (2020).

55. D. Ma, X. Li, Y. Guo, J. Chu, S. Fang, C. Yan, J. P. Noel, H. Liu, Cryptochrome 1 interacts with PIF4 to regulate high temperature-mediated hypocotyl elongation in response to blue light. *Proc. Natl. Acad. Sci. U.S.A.* **113**, 224–229 (2016).

56. U. Alon, *An Introduction to Systems Biology: Design Principles of Biological Circuits* (Chapman and Hall, CRC Press, ed. 2, 2020).

57. S. N. Gangappa, S. V. Kumar, DET1 and HY5 control PIF4-mediated thermosensory elongation growth through distinct mechanisms. *Cell Rep.* **18**, 344–351 (2017).

58. K. Lau, R. Podolec, R. Chappuis, R. Ulm, M. Hothorn, Plant photoreceptors and their signaling components compete for COP1 binding via VP peptide motifs. *EMBO J.* **38**, e102140 (2019).

59. J. Ponnu, T. Riedel, E. Penner, A. Schrader, U. Hoecker, Cryptochrome 2 competes with COP1 substrates to repress COP1 ubiquitin ligase activity during *Arabidopsis* photomorphogenesis. *Proc. Natl. Acad. Sci. U.S.A.* **116**, 27133–27141 (2019).

60. B. Parent, F. Tardieu, Temperature responses of developmental processes have not been affected by breeding in different ecological areas for 17 crop species. *New Phytol.* **194**, 760–774 (2012).

61. A. Raschke, C. Ibañez, K. K. Ullrich, M. U. Anwer, S. Becker, A. Glöckner, J. Trenner, K. Denk, B. Saal, X. Sun, M. Ni, S. J. Davis, C. Delker, M. Quint, Natural variants of ELF3 affect thermomorphogenesis by transcriptionally modulating PIF4-dependent auxin response genes. *BMC Plant Biol.* **15**, 197 (2015).

62. T. Bu, S. Lu, K. Wang, L. Dong, S. Li, Q. Xie, X. Xu, Q. Cheng, L. Chen, C. Fang, H. Li, B. Liu, J. L. Weller, F. Kong, A critical role of the soybean evening complex in the control of photoperiod sensitivity and adaptation. *Proc. Natl. Acad. Sci. U.S.A.* **118**, e2010241118 (2021).

63. K. A. Hicks, T. M. Albertson, D. R. Wagner, EARLY FLOWERING3 encodes a novel protein that regulates circadian clock function and flowering in Arabidopsis. *Plant Cell* **13**, 1281–1292 (2001).

64. E. Huq, P. H. Quail, PIF4, a phytochrome-interacting bHLH factor, functions as a negative regulator of phytochrome B signaling in Arabidopsis. *EMBO J.* **21**, 2441–2450 (2002).

65. P. Leivar, E. Monte, Y. Oka, T. Liu, C. Carle, A. Castillon, E. Huq, P. H. Quail, Multiple phytochrome-interacting bHLH transcription factors repress premature seedling photomorphogenesis in darkness. *Curr. Biol.* **18**, 1815–1823 (2008).

66. J. W. Reed, P. Nagpal, D. S. Poole, M. Furuya, J. Chory, Mutations in the gene for the red/far-red light receptor phytochrome B alter cell elongation and physiological responses throughout Arabidopsis development. *Plant Cell* **5**, 147–157 (1993).

67. T. W. McNellis, A. G. von Arnim, T. Araki, Y. Komeda, S. Miséra, X. W. Deng, Genetic and molecular analysis of an allelic series of cop1 mutants suggests functional roles for the multiple protein domains. *Plant Cell* **6**, 487–500 (1994).

68. T. Oyama, Y. Shimura, K. Okada, The Arabidopsis HY5 gene encodes a bZIP protein that regulates stimulus-induced development of root and hypocotyl. *Genes Dev.* **11**, 2983–2995 (1997).

69. S. Lorrain, T. Allen, P. D. Duek, G. C. Whitelam, C. Fankhauser, Phytochrome-mediated inhibition of shade avoidance involves degradation of growth-promoting bHLH transcription factors. *Plant J.* **53**, 312–323 (2008).

70. R. Yamaguchi, M. Nakamura, N. Mochizuki, S. A. Kay, A. Nagatani, Light-dependent translocation of a phytochrome B-GFP fusion protein to the nucleus in transgenic Arabidopsis. *J. Cell Biol.* **145**, 437–445 (1999).

71. L. I. A. Calderon-Villalobos, C. Kuhnle, H. Li, M. Rosso, B. Weisshaar, C. Schwechheimer, LucTrap vectors are tools to generate luciferase fusions for the quantification of transcript and protein abundance in vivo. *Plant Physiol.* **141**, 3–14 (2006).

72. C. Grefen, N. Donald, K. Hashimoto, J. Kudla, K. Schumacher, M. R. Blatt, A ubiquitin-10 promoter-based vector set for fluorescent protein tagging facilitates temporal stability and native protein distribution in transient and stable expression studies. *Plant J.* **64**, 355–365 (2010).

73. M. Balcerowicz, K. Fittinghoff, L. Wirthmueller, A. Maier, P. Fackendahl, G. Fiene, C. Koncz, U. Hoecker, Light exposure of Arabidopsis seedlings causes rapid de-stabilization as well as selective post-translational inactivation of the repressor of photomorphogenesis SPA2. *Plant J.* **65**, 712–723 (2011).

74. S. Kirkpatrick, C. D. Gelatt, M. P. Vecchi, Optimization by simulated annealing. *Science* **220**, 671–680 (1983).

75. H. L. Hsieh, H. Okamoto, M. Wang, L. H. Ang, M. Matsui, H. Goodman, X. W. Deng, FIN219, an auxin-regulated gene, defines a link between phytochrome A and the downstream regulator COP1 in light control of Arabidopsis development. *Genes Dev.* **14**, 1958–1970 (2000).

76. C. Nieto, L. M. Luengo, S. Prat, Regulation of COP1 function by Brassinosteroid signaling. *Front. Plant Sci.* **11**, 1151 (2020).
